# Geometry of the Gene Expression Space of Individual Cells

**DOI:** 10.1371/journal.pcbi.1004224

**Published:** 2015-07-10

**Authors:** Yael Korem, Pablo Szekely, Yuval Hart, Hila Sheftel, Jean Hausser, Avi Mayo, Michael E. Rothenberg, Tomer Kalisky, Uri Alon

**Affiliations:** 1 Department of Molecular Cell Biology, Weizmann Institute of Science, Rehovot, Israel; 2 Department of Medicine, Division of Gastroenterology and Hepatology, and Institute for Stem Cell Biology and Regenerative Medicine, Stanford University, Stanford, California, United States of America; 3 Faculty of Engineering, Bar Ilan University, Ramat Gan, Israel; University of California San Diego, UNITED STATES

## Abstract

There is a revolution in the ability to analyze gene expression of single cells in a tissue. To understand this data we must comprehend how cells are distributed in a high-dimensional gene expression space. One open question is whether cell types form discrete clusters or whether gene expression forms a continuum of states. If such a continuum exists, what is its geometry? Recent theory on evolutionary trade-offs suggests that cells that need to perform multiple tasks are arranged in a polygon or polyhedron (line, triangle, tetrahedron and so on, generally called polytopes) in gene expression space, whose vertices are the expression profiles optimal for each task. Here, we analyze single-cell data from human and mouse tissues profiled using a variety of single-cell technologies. We fit the data to shapes with different numbers of vertices, compute their statistical significance, and infer their tasks. We find cases in which single cells fill out a continuum of expression states within a polyhedron. This occurs in intestinal progenitor cells, which fill out a tetrahedron in gene expression space. The four vertices of this tetrahedron are each enriched with genes for a specific task related to stemness and early differentiation. A polyhedral continuum of states is also found in spleen dendritic cells, known to perform multiple immune tasks: cells fill out a tetrahedron whose vertices correspond to key tasks related to maturation, pathogen sensing and communication with lymphocytes. A mixture of continuum-like distributions and discrete clusters is found in other cell types, including bone marrow and differentiated intestinal crypt cells. This approach can be used to understand the geometry and biological tasks of a wide range of single-cell datasets. The present results suggest that the concept of cell type may be expanded. In addition to discreet clusters in gene-expression space, we suggest a new possibility: a continuum of states within a polyhedron, in which the vertices represent specialists at key tasks.

## Author Summary

In the past, biological experiments usually pooled together millions of cells, masking the differences between individual cells. Current technology takes a big step forward by measuring gene expression from individual cells. Interpreting this data is challenging because we need to understand how cells are arranged in a high dimensional gene expression space. Here we test recent theory that suggests that cells facing multiple tasks should be arranged in simple low dimensional polygons or polyhedra (generally called polytopes). The vertices of the polytopes are gene expression profiles optimal for each of the tasks. We find evidence for such simplicity in a variety of tissues—spleen, bone marrow, intestine—analyzed by different single-cell technologies. We find that cells are distributed inside polytopes, such as tetrahedrons or four-dimensional simplexes, with cells closest to each vertex responsible for a different key task. For example, intestinal progenitor cells that give rise to the other cell types show a continuous distribution in a tetrahedron whose vertices correspond to several key sub-tasks. Immune dendritic cells likewise are continuously distributed between key immune tasks. This approach of testing whether data falls in polytopes may be useful for interpreting a variety of single-cell datasets in terms of biological tasks.

## Introduction

Recent advances allow high-throughput measurement of biological information from individual cells [1–12]. This is an improvement over standard experiments which mask the range of states in the population because they average over millions of cells. Therefore, it is expected that single-cell technologies can reveal new biology, such as the diversity of states of cells in a tissue [13–21]. These experiments portray each cell as a point in a high-dimensional space whose axes are the expression level of each gene, or other molecular parameters.

The geometry of how cells are distributed in gene expression space is an open question. One possibility is that each cell type forms a tight cluster, and that these clusters are well separated from each other. This assumption is at the heart of clustering analyses of gene expression data [22,23]. The tight-cluster picture relates to the idea of discrete cell types, which is supported by the existence of marker genes that are mutually exclusive between cells. When considering a set of many genes, in contrast to only marker genes, it is possible that cells also form more continuous distributions in gene expression space, and that clusters are more difficult to define. Such distributed states have been suggested in studies on T-cells [24–26] and embryonic stem cells [27]. In such cases, an open question is whether there is meaningful geometry to this continuum of cell states.

The question of geometry in gene expression space was recently addressed in the context of a theory on evolutionary tradeoffs [28]. The theory suggests that cells that need to perform multiple tasks are arranged in a simple, low dimensional polygons or polyhedra in gene expression space. The vertices of these shapes, called archetypes, are the expression profiles optimal for each task. Thus, two tasks correspond to data on a line, three tasks to data on a triangle, four tasks to a tetrahedron, and so on. These shapes are generally called polytopes: the generalization of polygons and polyhedral to any number of dimensions. These polytopes represent the optimal tradeoffs between the tasks, in the sense that for any point outside the polytope there is a point inside it that performs equally or better at all tasks. This corresponds to the concept of Pareto optimality, where the polytopes are Pareto fronts for the system [28–32]. Pareto optimality has been applied to a variety of biological datasets [30,33–36], but not to single-cell data.

**Fig 1:**
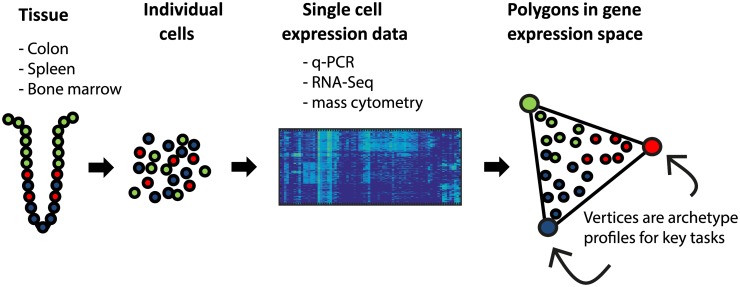
Overview of Pareto archetype analysis of single-cell datasets to discover polytopes in gene expression space and infer tasks. Datasets from different human and mouse tissues analyzed by different groups with different technologies were analyzed by Pareto archetype analysis. Best fit polytopes and their significance were found. Tasks were inferred from the genes maximally enriched in the cells closest to each vertex of the polytope.

Here, we study single-cell expression from several tissues, collected with different single-cell technologies (Fig 1). We analyze single-cell qPCR data on human and mouse colonic crypts from Dalerba et al. and Rothenberg et al. [2,37], single-cell mass cytometry data on individual human bone marrow cells from Bendall et al [13,25], and single-cell RNA-Seq on mouse dendritic cells from Jaitin et al [3]. We test whether each dataset is well-described by a low dimensional polytope. We fit the data to a series of polytopes (line, triangle, tetrahedron, etc.), finding the best fit polytope, and assess its statistical significance. We then analyze the gene expression profiles at the cells closest to the vertices (archetypes), to test if they correspond to specific biological tasks. This offers a way to discover potential tasks of cells in a tissue from single-cell data.

We find evidence for polytopes and enriched tasks in all of the datasets we analyzed. The well-studied human and mouse colonic crypt system [38,39] includes stem cells at the bottom of the crypt, which differentiate into enterocytes that absorb nutrients, and secretory cells, mainly goblet cells, which secrete mucus. We find that single human intestinal cells are arranged in gene expression space, to a good approximation, in a tetrahedron. At its vertices are expression profiles of pure cell types—enterocytes, goblet cells, putative stem cells and a new category of nodal-expressing cells. We further find that when analyzing the intestinal progenitor cells alone, they fill out a distinct tetrahedron in gene expression space, in contrast to other cell types. The vertices of this tetrahedron correspond to four progenitor cell tasks. In addition, we find that the polytopes found in crypt data from human and mice are strikingly similar. Using the same approach, we find that human bone marrow cell data is arranged in a five-vertex simplex (four-dimensional simplex) whose vertices correspond to five major cell types, and that mouse dendritic cells uniformly fill a tetrahedron suggesting four immune tasks including maturation, pathogen sensing and communication with lymphocytes. Pareto analysis can thus be useful to understand the geometry of single-cell gene expression, and to infer the tasks of single cells in a tissue.

## Results

### Pareto analysis on human crypt single-cell expression suggests four archetypes

We begin with analyzing the single-cell gene expression dataset of human colon crypt cells obtained by Dalerba et al [2]. The dataset included 407 individual cells, each analyzed by single-cell qPCR for 83 selected genes in a Fluidigm microfluidic system [5]. We normalized the data by subtracting the mean of each gene as described in Methods.

To address the effects of outliers and all/none bimodality in expression data ([11,12]), we removed 10% of the cells which had the lowest expression levels across genes, and 10% of the genes which had the lowest expression across cells, resulting in a dataset of 368 cells and 76 genes (S1 Dataset). Removing more cells or genes, up to 25% of the lowest expressing cells or lowest expressed genes, leaves the results essentially unchanged (S1 Fig and S1A Text).

Based on theory on evolutionary tradeoffs between tasks [28], we expect that cells should fall in a low-dimensional polytope whose vertices are the points optimal in each task alone. We therefore asked whether gene expression data is enclosed within a low dimensional polytope (e.g. line, triangle, tetrahedron and so on). We calculated the best fit polytopes that enclose the data, and asked how well they describe the data compared to randomized datasets. We considered polytopes with k vertices: we tested k = 2 (line), k = 3 (triangle), k = 4 (tetrahedron) and so on up to 11 vertices. The polytopes were found using the PCHA algorithm [40]. This algorithm seeks k points on the convex hull of the data that define a polytope that encloses as much of the data as possible (see Methods: Archetype detection using the PCHA algorithm [40]).

**Fig 2:**
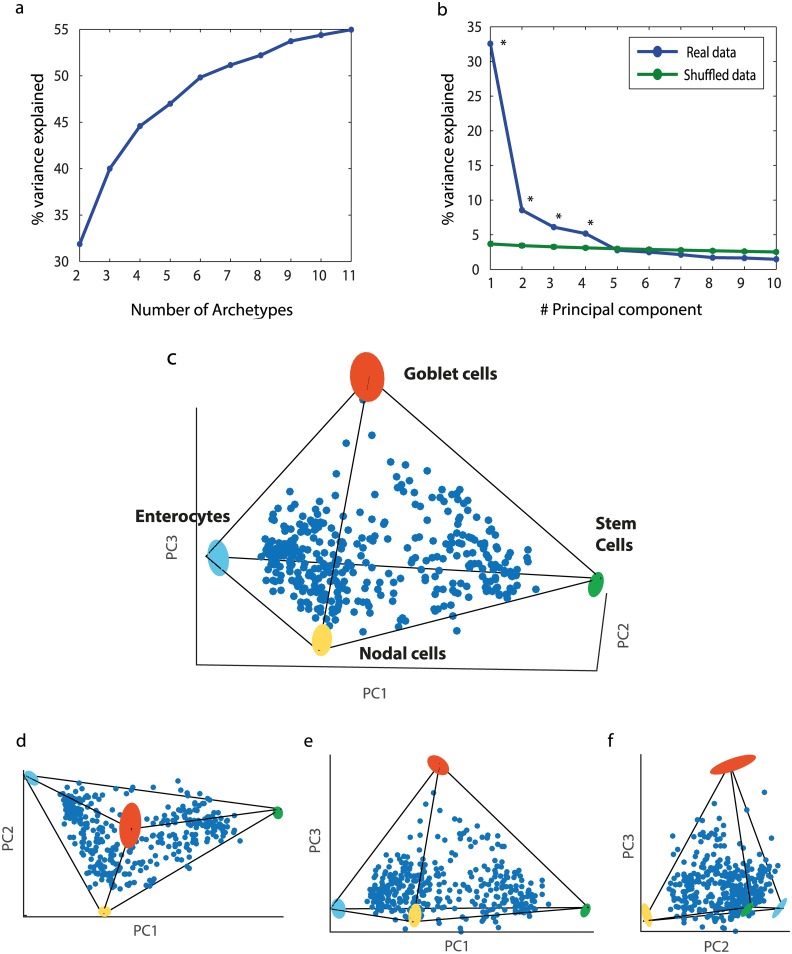
Human colon crypt cells fall in a tetrahedron in gene expression space. (a) For k = 2–11 we found the k-polytope that best fit the data using PCHA algorithm, considering all 76 dimensions. Explained variance of best fit polytopes with k = 2–11 vertices begins to saturate at k = 4 or k = 5 vertices. (b) Comparison between the variance explained by the first k principal components of the data to the variance explained by the k principal components of shuffled data suggests that effective data dimensionality is three or four. Blue line: variance explained by PCA of intestinal data. Green line: variance explained by PCA of shuffled data. Points represent mean values. Error bars, representing 5%-95% variation intervals, are smaller than line width. Points for which the real data EV is higher than the randomized data EV are marked with *. (c) Data displayed in first 3 PCs axes resembles a tetrahedron, and its projections on principal planes (d)-(f) resemble triangles. Archetypes and their variation upon data resampling (bootstrapping) are shown as colored ellipses (see S1B Text). Thin lines—tetrahedron edges.

For each polytope, we calculated the deviation of the data from the polytope (explained variance, Methods: Determining the number of archetypes). k = 4 archetypes explain 45% of the variance, whereas adding a fifth archetype added only 4% additional explained variance (Fig 2a). A tetrahedron explained the data variance better than when applying the same algorithm to shuffled data (p = 0.01, Methods: Statistical significance of best fit polytopes). This suggests that a 4-vertex polyhedron, namely a tetrahedron, is a reasonable description of the data.

The tetrahedron was found in the full 76-dimensional space. To display the data, it is helpful to use principal component analysis (PCA). PCA indicated that 3 principal components (3 PCs) explain most of the variance in the data (47%), and that 4 PCs (52%) are significant compared to shuffled data (Fig 2b, Methods: Determining the number of archetypes), indicating that the data is indeed low-dimensional. The tetrahedrality of the data is highlighted by the fact that 96% of the variance explained by the first 3PCs is explained also by the much more stringent description of a tetrahedron whose vertices are on the convex hull of the data. Plotting the data, with each cell represented by a point in the space spanned by the first 3PCs of gene expression space, suggests a tetrahedron-like shape (Fig 2c). The projections of the data on the three principal planes are roughly triangular (Fig 2d–2f), and show well-defined linear edges which meet at pointy vertices.

We found that the archetype positions were robust to data sampling, with errors on the order of a few percent in bootstrapping tests in which data is resampled with replacement (S1B Text and S1a Table and Fig 2c). We note that clustering methods, such as k-means or hierarchical clustering, are much more sensitive to sampling errors in this dataset: the continuous distribution of the data makes the cluster boundaries somewhat arbitrary and thus on the order of 20% of the data points are classified to different clusters upon bootstrapping (S1C Text and S2–S4 Figs).

### Each archetype is enriched with markers for a major crypt cell type

**Fig 3:**
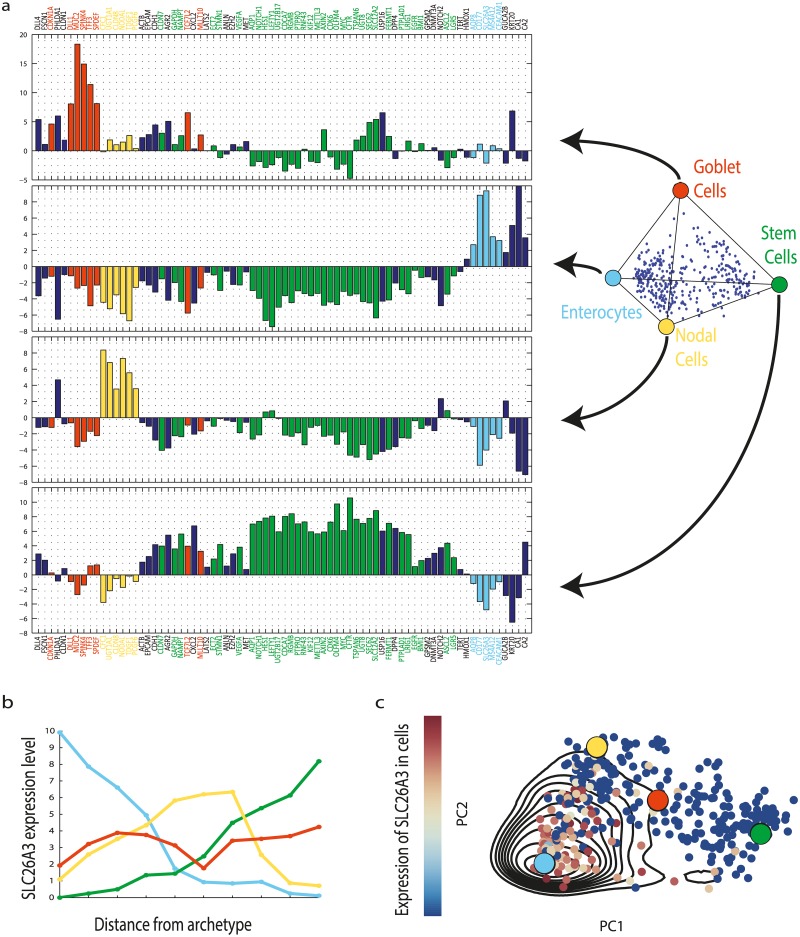
Expression profiles of the four colon crypt archetypes are each enriched for markers of specific cell types. (a) The expression profiles of the four archetypes, with enriched genes colored. Enriched genes were determined by leave-1-out enrichment analysis, binning the cells according to distance from each archetype and seeking when average expression in the bin closest to the archetype is maximal, as described in Methods: 1D Gene enrichment at archetypes (See full enriched genes list in S2 Table). Light blue—enterocyte archetype, yellow—Nodal archetype, green—stem cells archetype, red—goblet cell archetype. Genes that are not enriched, or enriched in more than one archetype, are in dark blue. Zero level represents the average expression of each gene in the dataset. (b) Leave-1-out enrichment plot: expression of a gene (SLC26A3—an enterocyte marker) as a function of distance from archetype in equal mass bins of cells (Methods: 1D Gene enrichment at archetypes), line color indicates archetype. This gene is maximally enriched only at the enterocyte archetype (blue line). For enrichment plots for additional genes see S5 Fig. (c) A two dimensional enrichment plot of SLC26A3, in which its expression is plotted on the plane of the first 2PCs of the data, indicating expression is maximal in the cells closest to the enterocyte archetype. Contours are expression density estimated using a Gaussian kernel (Methods: 2D Gene enrichment at archetypes). Archetype positions and PCs were calculated without the tested gene.

Each of the four vertices of the best-fit tetrahedron is a point in the 76-dimensional gene expression space. Within Pareto theory, each vertex is an archetype that can be thought of as an optimal gene expression profile, extrapolated from the data, which best performs the archetype's task. The gene profiles for the archetypes are shown in Fig 3a. We find that each of the four archetypes shows high expression of a set of markers for a specific crypt cell type (Fig 3b and 3c and S5 Fig).

Archetype 1 shows high expression of enterocyte markers (AQP8, SLC26A3, MS4A12, KRT20 [41–44]). The cells closest to this archetype show the maximal expression of these markers in the entire dataset (Methods: 1D Gene enrichment at archetypes). In cells closest to archetype 2, putative stem cells markers are maximally expressed (including LGR5, ASCL2, AXIN2, c-MYC, CDK6, OLFM4 [45–49]). In cells closest to archetype 3, markers for goblet cells are maximally expressed (MUC2, TFF3, SPDEF and others [38,50,51]). The complete list of enriched genes is shown in S2 Table. Thus, the first three archetypes correspond each to one of the three main crypt cell types. The fourth archetype is discussed below.

We also evaluated the physical position of each cell in the crypt using a proxy for height in the crypt, Axin2 expression [52]. We find that the stem cell archetype has highest Axin2, followed by the goblet archetype. The enterocyte archetype has lowest Axin2. This matches the known arrangement of cells in the crypt, with stem cells at the bottom of the crypt, and enterocytes at its top (S6a Fig) [37].

### One archetype represents a novel Nodal cell class which may be an intermediate between stem cells and enterocytes

The fourth archetype is enriched with a specific set of genes related to development and embryonic patterning (NODAL, CFC1, TDGF1 [53–56]), a transcriptional repressor that has a role in development (PCGF6 [57]), and an enzyme involved in hormone secretion (UGT1A1 [58]), and a member of the claudin family CLDN8 [59]. We call these cells Nodal cells. Their position in the crypt, based on Axin2 levels, is intermediate between the bottom and top (S6a Fig).

To better understand Nodal cells, we ordered the cells according to a pseudo-temporal order inferred from their gene expression using the Wanderlust algorithm [60]. NODAL cells seem to lie on the developmental axis between stem cells and enterocytes (S7a Fig). This tentatively suggests NODAL cells as a differentiation step between precursors and enterocytes.

In summary, Pareto analysis finds four archetypes which correspond to gene expression profiles. Three of these define the three main cell types in the crypt. The fourth may indicate a step between stem cells and enterocytes. The archetypes can be interpreted as an idealized gene profile for each of the cell types, and cells of a given type are arranged in proximity to the corresponding archetype in gene expression space.

### Progenitor cells uniformly fill a tetrahedron with archetypes for stemness and three differentiation tasks

**Fig 4:**
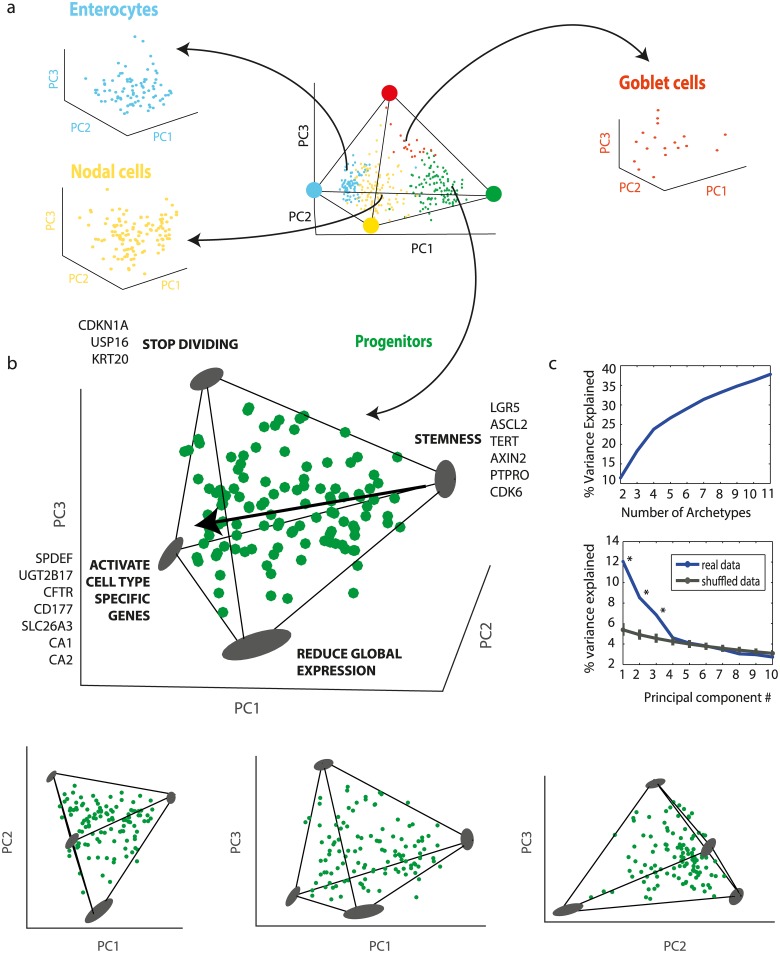
Progenitor crypt cells fall in a tetrahedron. (a) Enterocytes, goblet cells and nodal cells analyzed separately do not form significant polytopes. Cells are color coded by type in the tetrahedron of Fig 3, and each cell class is plotted in its own first 3PC. (b) Progenitor cells analyzed separately fall uniformly in a tetrahedron. The best fit tetrahedron is shown (PCHA delta = 0.5). Arrow represents direction of development according to Axin2 levels, see S7b Fig. Also shown are projections on the principal planes, which resemble triangles or quadrangles. Archetypes and their variation upon data resampling (bootstrapping) are shown as gray ellipses. (c) Explained variance as a function of polytope order k or number of PCs D both suggest a tetrahedron (k = 4, D = 3).

We next zoom in on each inferred cell type to examine the variation between cells within a type. We repeated the Pareto analysis on each class of cells separately (S1D Text). We find that the three non-progenitor cell types (enterocytes, goblet cells, nodal cells) cannot be explained by a statistically significant polytope with 5 or less vertices (see S3 Table, all p-values >0.15). Thus, the expression of these cells seems to form a cloud in gene expression space with no easily discernible vertices (Fig 4a). This may hint that other effects dominate the structure of the data, or that not enough cells or not enough relevant genes were measured, see S1E Text and S8 Fig.

In contrast, the progenitor cells were well-described by a tetrahedron (p = 0.01, Methods: Statistical significance of best fit polytopes, Fig 4b showing tetrahedron and projections, Fig 4c shows explained variance curves, S2 Dataset). The progenitor cells seem to uniformly fill out this tetrahedron, suggesting that precursors span a continuum of gene expression states, with some cells coming close to one of four archetypal precursor profiles, and others showing a more even mixture of the archetypes.

We examined the expression profiles of the cells closest to each archetype (Methods: 1D Gene enrichment at archetypes). Archetype 1 is enriched with stem markers LGR5 and ASCL2, and lies physically at the lowest point in the crypt according to Axin2 levels (S7b Fig inset). It has proliferation markers (MYC, Cdk6), and telomerase (TERT). These genes are characteristic of dividing stem cells [37,46,48,49].

The other three archetypes all display the progenitor marker OLFM4 [61], but also have characteristics more similar to the differentiated cells. Archetype 2 includes enrichment in enterocyte and goblet markers. Archetype 3 is enriched in division inhibitor CDKN1A [62]. Archetype 4 has low expression of all genes. We hypothesize that these three archetypes represent three tasks needed for differentiation: (i) expression of effector cell-specific genes (ii) inhibition of cell division (iii) reduction in global gene expression. For a complete list of enriched genes see S4 Table, archetype gene expression profiles are shown in S9 Fig.

The progenitor cells fill out the tetrahedron quite uniformly. According to Axin2 expression, as they move up the crypt they move away from the stem archetype and parallel to the plane defined by the other three archetypes. Pseudo-temporal order derived by the Wanderlust algorithm suggests similar conclusion (S7b Fig). This may suggest multiple temporal paths between the three tasks, such that each progenitor is a different weighted average of the archetypes.

We also asked how the progenitor tetrahedron relates to the rest of the cells in the crypt. We find that the fully differentiated cells types (enterocytes, goblet cells) are closest to archetype 4, because they all have lower overall gene expression than the progenitors.

### Similar polytopes for mouse and human intestinal crypt single-cell data

Up to now we analyzed a human crypt dataset. We now compare it to a mouse crypt dataset, presented by Rothenberg et al. [37] using qPCR (S3 Dataset). We used the same Pareto analysis approach. We removed the lowest 10% of cells and genes, remaining with 161 cells and 41 genes. The two datasets overlap in 24 genes. The mouse cells in the dataset were only from the bottom of the crypt: they were harvested by cell sorting (FACS) using the markers CD66 and CD44.

**Fig 5:**
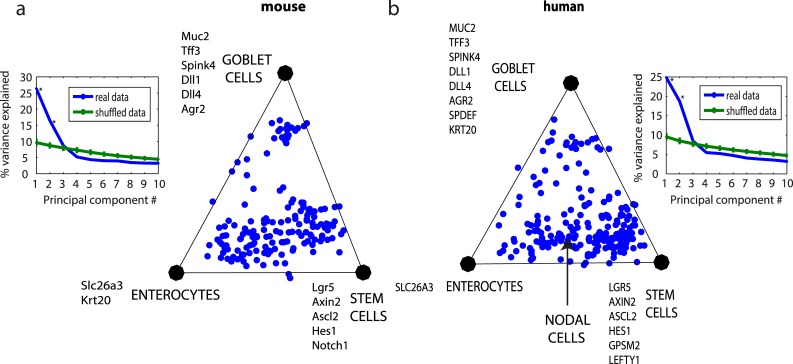
Mouse and human colon lower crypt cells fall on similar triangles and show a similar distribution within the triangle. (a) Mouse colon lower crypt cells dataset by [37], plotted on its first 2PCs plane. Inset: PCA explained variance analysis suggest k = 3 vertices and D = 2 dimensions, namely a triangle. (b) Human lower crypt dataset plotted on its first 2PC plane. Inset: explained PCA explained variance analysis. The 24 genes common to the two datasets were used. Arrow indicates projection of the nodal cell archetype on the triangle.

We find that the mouse single-cell data is well-described by a triangle (p = 0.003, 2PCs explain 43% of the variance). To compare the mouse data to human data described in the previous sections, we analyzed human cells in the lower part of the crypt (defined in [2], by FACS sort for high and low cells using CD66 and CD44 markers). This dataset (213 cells) is also well described by a triangle (p = 0.001, 2PCs describe 35% variance). The archetypes in both the datasets correspond to stem cells, goblet cells and enterocytes. The nodal cells are on the edge connecting progenitors and enterocytes (Fig 5b), representing the projection of the nodal tetrahedral archetype on the triangle that describes the crypt-bottom cells. In the mouse dataset 5 out of the 6 genes that are enriched in the Nodal archetype were not measured; however the Nodal co-receptor TDGF1 [27] is highly expressed on the edge that connects stem cells and enterocytes.

The geometry of mouse and human datasets are strikingly similar (Fig 5). The three mouse archetypes are very similar to the three corresponding human archetypes in their overlapping genes (R^2^ = 0.65, p<10^–9^, S10 Fig, enriched genes are shown in Fig 5), and signify stem cells, goblet cells and enterocytes. Moreover, the distribution of points on the triangle suggests a gradual differentiation process from stem cells to enterocytes, compared to more abrupt temporal switch in the case of differentiation to goblets, a difference shared by both species.

We also analyzed single-cell data by Dalerba et al [2] for human colon cancer xenograft in mouse, derived from a single cancer cell (S11 Fig and S1F Text and S4 Dataset). We compared this data to the triangle found when analyzing human crypt-bottom normal tissue cells (Fig 5b). We find that the cancer cells lie in a similar triangle, with a density distribution that peaks near the stem cells, enterocyte and goblet archetypes. This hints that the human cancer cells undergo differentiation similar to the normal mouse and human tissues [2].

### Analysis of single-cell mass-cytometry of bone marrow cells and RNA-Seq of dendritic cells reveals polytopes and tasks

**Fig 6:**
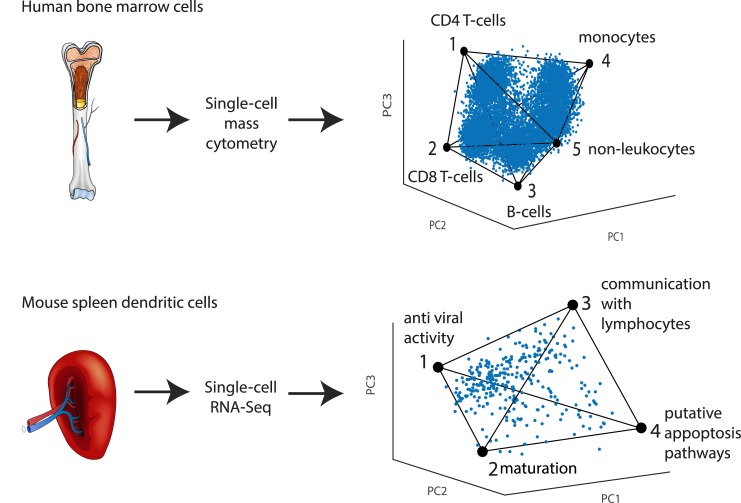
Different tissues analyzed by different single-cell technologies show polytopes and tasks. (a) Human bone marrow cells analyzed by single-cell mass cytometry [13,25] in which proteins are detected using mass-tagged antibodies is well described by a 4D simplex (a polytope with 5 vertices). The simplex is shown projected on the first 3PCs, for other projections see S12c Fig. The archetypes correspond to cell types as indicated. Cell density peaks near each archetype. (b) Mouse spleen LPS stimulated dendritic cells analyzed by single-cell RNA-Seq [3] are well described by a tetrahedron. Archetypes are labeled with functions inferred from genes maximally enriched in cells near each archetype. For more details see S1G and S1H Text.

Finally, we asked whether this approach can be used with other experimental techniques and other tissue types. We studied a single-cell mass cytometry (Cytof) dataset on human bone marrow by Bendall et al [13,25] (S5 Dataset). Here, 10,000 cells were each characterized by the expression of 31 proteins detected using antibodies. We find that this dataset is well-described by a four-dimensional simplex (a polytope with five vertices, p = 0.005, Fig 6 top row and S12 Fig). The five archetypes are each enriched with genes that clearly define a specific cell type (CD4 T cells, CD8 T cells, monocytes/ macrophages, B cells and non-leukocytes) (see S1G Text and S5 Table and S13 and S14 Figs). Cells density peaks near each vertex. A sizable set of cells, which formed an unidentified cluster in the viSNE analysis of [13], lies in the middle of the tetrahedron (S15 Fig), possibly indicating cells whose protein expression is intermediate between the classical cell type profiles.

We also analyzed a single-cell RNA-Seq dataset from mouse spleen by Jaitin et al [3] (S6 Dataset). Each cell is characterized by 20,091 gene expression counts, based on sampling a fraction of the cell’s mRNA pool. This data was classified by Jaitin et al into seven groups of cells using a probabilistic mixture model. One group of cells, however, seemed to defy clear clustering (class VII in [3]). These are the dendritic cells in the spleen, known to carry out a wide range of immune functions including detection of pathogens and activation of lymphocytes [63–65]. We find that LPS treated dendritic cells in this dataset are well-described by a tetrahedron (312 cells, p<0.001, Fig 6 bottom row). Several functional gene groups [66] (see S1H Text) are highly and maximally enriched in the cells closest to each of the four archetypes (S6 Table). This enrichment indicates four key immune tasks: Archetype 1- response to virus (cytoplasmic DNA response and interferon pathways) [7,67]; Archetype 2- Dendritic cell maturation and formation of cytoskeletal features [64,68,69]; Archetype 3- Stimulation of lymphocytes (cytokine secretion, antigen presentation) [7,70,71]; Archetype 4- putative cell-death pathways [72,73].

We compared the tissues in terms of how uniformly they fill out their polyhedra or polytopes. We therefore computed the ratio *ρ* between the mean local density of the data and the local density expected in a uniform distribution [74] (S1I Text and S16 Fig). The closer this ratio is to one, the more uniform the distribution of points; a high ratio means the data is clumped into clusters. We find that bone marrow cells are the most clustered or clumped among the datasets (*ρ* = 4.67), in line with the classic view of hierarchical hematopoietic lineages [75,76]. Intestinal cells are less clustered (*ρ* = 2.93). The closest to uniform distribution inside their respective polyhedra are dendritic cells (*ρ = 2*.*42*) and intestinal progenitors (*ρ* = 1.06).

We conclude that the present approach can describe the geometry and potential tasks of single-cell data from diverse tissues and different technologies.

## Discussion

We studied the geometry of single cells in gene expression space using Pareto archetype analysis. We used data from different single-cell technologies employing qPCR, RNA-Seq or mass cytometry, and different tissues including intestinal crypt, bone marrow and spleen. We find that single-cell data fall in low dimensional shapes with well-defined vertices, such as tetrahedrons. The cells closest to each vertex are enriched with genes that reveal key biological functions relevant to each tissue. Some datasets fall into distinct clusters, with one cluster near each vertex, and thus support a picture of distinct and well-separated cell types. Other contexts, such as intestinal progenitor cells and spleen dendritic cells, show a continuum of gene expression states which uniformly fills the tetrahedron, supporting a picture of a continuous range of cell states that carry out mixtures of the biological functions defined by the vertices. These findings expand the concept of cell type, by demonstrating the possibility of a polyhedral continuum of expression states: cells can range between being task specialists near the vertices of the polyhedron, and generalists suitable for multiple tasks near the center.

It is interesting to ask when is it better to design distinct cell types with separated biological functions, and when to design a continuum of cell expression states. Distinct cell types have the advantage of being specialists at a given task, with optimal function. However, if the proportions of the tasks needed in the tissue changes more rapidly than the ability to make new cells or to adjust their protein composition, a continuum of states may have an advantage. It allows a reserve of cells (cells in the middle of the polyhedron) that can perform multiple functions, albeit less optimally than specialists, and can therefore be recruited to each task in times of need.

This type of reasoning was used to explain why ant morphologies in a nest tend to show a continuous distribution rather than distinct clusters: Intermediate morphology ants can be recruited to defense, foraging or nursing tasks according to changing needs [77]. Other factors that may influence clustering versus continuum include the biochemical feasibility of multiple functions to co-exist in the same cell [78,79] and the existence of a continuous range of spatial and temporal niches in a tissue related for example to migration, differentiation processes [17,80], or to distances from blood vessels or tissue boundaries [81].

We find a continuous filling of a tetrahedron in the context of progenitor cells in the intestine. The progenitor tetrahedron suggests four tasks—one related to stemness and stem cell renewal, and the other three archetypes related to potential tasks required for early stages of differentiation: stopping division, expressing effector genes, and down regulating global expression [82]. The uniform filling of the progenitor tetrahedron suggests that there is not one temporal path to differentiation, but rather many paths with different ordering of the tasks, each taken with more or less equal probability. This relates to the idea that stem cells show heterogeneity [15,27,83], in which different molecular states confer functional biases to individual cells, contributing to their overall regulation [84], and aligns with recent findings about their plasticity [85,86]. With more data, one may be able to infer temporal paths from static data, as was done in the context of the cell cycle [87] and cell differentiation [17,60].

Similarly, the continuum observed between intestinal progenitors, nodal cells and enterocytes (Figs 2c and 5b) suggests a gradual differentiation process, with the nodal cells—a new class of cells defined in the present study—possibly an intermediate station between progenitors and enterocytes. The polyhedral continuum of states we find in stimulated dendritic cells (DCs) may likewise suggest spatiotemporal trajectories in the spleen, with external DCs active in pathogen detection, followed by migration into the central spleen for lymphocyte activation [64,65].

The present Pareto analysis is a new way of looking at single-cell data that emphasizes the geometric contours that enclose the data. This approach was used recently also in other biological contexts, to understand *C*. *elegans* foraging behavior [34], bird toe-bone proportions [35], and bacterial [88] and tumor [33] population-level gene expression. It is useful to compare the present approach to other methods of analyzing high-dimensional data, such as clustering or self-organizing maps [88–92]. If the data is arranged in separated non-overlapping clouds, all methods can reveal its structure. If the data, in contrast, is spread more along a continuum, clustering methods can lead to arbitrary classification, because it is not possible to tell where one cluster ends and another begins. For example, in the current crypt dataset, cluster assignment for 20% of the crypt cells changed upon data resampling with returns (bootstrapping, see S1C Text). In contrast, archetypes varied by only a few percent upon bootstrapping, and data description as a weighted average of archetypes was likewise much less sensitive to data resampling than clustering analysis (S2–S4 Figs and S1 Table).

The present approach also differs from principal component analysis (PCA, see S1J Text): whereas PCA finds the axes of the space which describes most of the data variance, our approach finds a polytope within which the data resides and is thus much more restrictive (S17 Fig); moreover the vertices of the polytope are different from the principal components (e.g. a tetrahedron resides in a 3D space but has four vertices, S18 and S19 Figs), and our findings support their interpretation as archetypal profiles that reveal clear biological functions. PCA and other dimensionality reduction techniques are not needed to find the polytopes, but can help to visualize them.

Major challenges remain, related to the high dimensionality of gene expression data, which can be in the many thousands. Fitting such data to a low-dimensional polytope can capture major trends. However, if there are small sets of genes that vary independently of the others, in a biologically important way, the current implementation of Pareto analysis will miss them because they make a small contribution to the overall shape of the data. Future work can develop ways to detect such groups of genes, separate the data and analyze each subgroup independently. This may lead to a presentation of the data as a collection (outer product) of Pareto fronts, each with a subset of genes related to a distinct set of tasks. Similarly, Pareto analysis can be used as a microscope to zoom in on a subset of cells or genes—in the present study, progenitor cells when considered separately without the rest of the crypt cells, revealed an informative tetrahedron of their own. Finally, one can analyze polytopes with increasing number of vertices and in this way observe the splitting of archetypes into finer distinctions (S1K Text and S20 Fig).

The polyhedra found here seem to be a distinct feature of the dataset, especially the straight edges and faces apparent in plots of the data distribution. However, it is possible that the observed polytopic-like structure results from other (unknown) reasons, such as systematic experimental errors or biological phenomena. Further application of Pareto analysis, with different tissues and different single-cell technologies that have different noise and biases, is needed to test the generality of the present conclusions. With these caveats in mind, the present approach of using Pareto analysis for single-cell data can be generally used to understand the geometry of single-cell data and to infer the tasks of individual cells in a tissue. More generally, this study indicates that the concept of cell type may be expanded. In addition to separated clusters in gene-expression space, we suggest a new possibility: a continuum of states within a polyhedron, in which the vertices represent specialists at key tasks, with generalist cells lying in the middle.

## Methods

### Preprocessing and normalization of single-cell qPCR data

Single-cell gene expression of primary human colon crypt cells obtained by Dalerba et al [2] included 407 individual cells, each analyzed by single-cell qPCR for 83 selected genes in a Fluidigm microfluidic system [5] (S7 Dataset). Note that the present dataset contains 34 genes not included in the original publication, kindly provided by Dalerba et al. These genes, including CFC1, UGT1A1, CLDN8, NODAL, TDGF1, and PCGF6, allow identification of the nodal cell type. Some genes were measured by multiple primers, see S1A Text and S21 Fig. Cells were sorted by FACS so that they belong to one of two populations: EpCAM^high^/CD44^+^, which corresponds to the bottom of the crypt, and EpCAM^+^/CD44^-^/CD66^high^, which corresponds the top of the crypt. The measured genes were selected using publicly available gene-expression array data sets from human colon epithelia, using a bioinformatics approach designed to identify developmentally regulated genes [2]. Gene expression was measured in Cycle threshold units (Ct)—the number of amplification cycles it takes to detect the gene—which correspond to the logarithm of the fold change in expression. The data was bi-modal, as seen in other applications of this technology [11,12], with some cells ranging from 2.5Ct to 30Ct, and another set of cells with Ct>40. Since there were no values larger than 30Ct, we treated this as the minimal threshold level of detection and set these values to 30, and then linearly transformed the data so that minimal CT is zero (30-data). Finally, we subtracted the mean of each gene. Cells and genes with low expression were removed as described in Results. The processed data can be found in S1 Dataset. The same normalization was used for the cancer xenograft data [2] (S4 Dataset) and for the mouse intestinal cells data (S3 Dataset) [37].

### Preprocessing and normalization of single-cell mass cytometry data

We used data by Bendall et al [25] and Amir et al [13], of human bone marrow samples from healthy donors. The cells were analyzed using single-cell mass cytometry as described in [25], resulting in the antibody-detected expression of 31 proteins in 10,000 cells. Multiple detections of one protein—CD3—were united by taking their mean for each cell. Following the original publication, we transformed the data by applying a hyperbolic arcsin transformation with a cofactor of five. Processed data can be found in S5 Dataset.

### Preprocessing and normalization of single-cell RNA-Seq data

We used data from [3], of mice spleen CD11c+ cells that were exposed to lipopolysaccharide (LPS) for 2 hours. We focused on cells that were classified in the original paper as dendritic cells (group VII). We used the likelihood model of [3] in order to choose cells which have the highest likelihood to belong to this group (rather than to other groups). We didn’t consider 16 genes with high batch-to-batch variability (as in [3]), and removed measures of control ERCC molecules. Since the data is very sparse (97% of the matrix entries are 0), we considered the 500 genes with highest standard deviation across samples. Results are similar for choosing genes by highest mean or median. Following Jaitin et al, we normalized the data by down-sampling: Defining a target number of molecules N, and then sampling from each cell having m> = N molecules precisely N molecules without replacement. Cells with m<N are not used for analysis. We used N = 400, and consequently considered 312 cells in our analysis. We then transformed the data by adding 1 and applying log_2_, in order to examine fold changes in expression, and reduced the average expression of each gene in all 312 cells. Processed data can be found in S6 Dataset.

### Archetype detection using the PCHA algorithm

We found the archetypes of the best fit polytope using the PCHA algorithm [40]. This algorithm finds the best fit polytope whose vertices are on the convex hull of the data. It does so by constraining the vertices to be a weighted average of the data points, where the weights are given by a matrix C, and then approximating the data points by a weighted average of the archetypes, where the weights are given by a matrix S. The algorithm then solves the following optimization problem using a projected gradient procedure:

$$\text{arg}\underset{C,S}{\text{min}}{\left\|X-XCS\right\|}_{F}^{2}$$

$$s.t.\;\left|C_{d}\right|\;=\;1,\;\left|S_{n}\right|\;=\;1$$

C≥0, S≥0

where X is the data matrix. The algorithm allows a relaxation of the optimization problem by introducing a parameter *δ* and relaxing the constraint on C to be

$$1-\delta \leq \left|C_{d}\right|\leq 1+\delta$$

This relaxation allows archetypes to be found within a certain volume around the data convex hull. Unless stated otherwise, we used *δ = 0*.*5* for archetypes characterization, polytope visualization and enrichment analysis, and *δ = 0* for explained variance calculation.

### Determining the number of archetypes

To determine the number of archetypes that describes the data, we computed the explained variance of the data (EV) for each number of archetypes (k = 2,3,4…k_max_). The explained variance is computed by PCHA as [88,93]:

$$EV\;=\;\frac{1}{N}\sum_{n\;=\;1}^{N}\left(1-\frac{\left|p_{n}-s_{n}\right|}{\left|p_{n}\right|}\right)$$

Where p_n_ is the n^th^ data point out of N points and S_n_ is the closest point to p_n_ in the polytope, and | denotes Euclidean distance. Then, we identify a number of archetypes k* for which adding an additional archetype does not increase EV by much (see Fig 2a). Operationally, k* was determined by the bend (also called elbow) in the EV versus k curve, defined by taking the most distant point from the line that passes through the first (k = 2) and the last (k = k_max_ = 11) points in graph. Changing k_max_ in the range 6–16 did not change the results for k* on the present datasets. We find for the present datasets that most polytopes beyond k* show low p-values (p<0.01), precluding the concern of type II errors (multiple hypothesis testing).

As a second indication for the order of the best fit polytope is the effective dimensionality of the data (e.g. a tetrahedron suggests that the data is effectively 3D). We therefore tested data dimensionality using Principal Component Analysis (PCA) [94]. We compared the variance explained by each PC to that of randomly shuffled data. Randomized data was created by shuffling the values of each coordinate, to preserve the density distribution of each coordinate while breaking correlations between coordinates. PCA on random data yields non-equal eigenvalues, due to stochastic correlations. Data dimensionality was found by detecting the point where the explained variance of the real data comes within a standard deviation of the shuffled data, (see Fig 2b). Data that is explained well by a k-vertex polytope is expected to have dimensionality of k-1 (e.g. data explained by a tetrahedron with k = 4 is essentially 3D).

### Statistical significance of best fit polytopes

Statistical significance of polytopes was tested by computing the EV of the real data compared to 1,000 sets of shuffled data, produced by randomly permuting each coordinate of the data separately. The explained variance (EV) by the PCHA algorithm as described above. The p-value was defined as the fraction of shuffled data sets for which the EV was larger or equal to the EV of the real dataset.

To make sure the low p-value stems from the similarity of the data to a polytope and not merely from its low dimensionality, we also performed PCA on the data and checked how similar it is to a polytope when projected to the space spanned by the first n-1 PCs (where n is the number of archetypes). A measure for the similarity of the data to a polytope is its t-ratio [28], defined as the ratio of the volume of convex hull of the data to the volume of the best fit polytope (we use PCHA with δ = 0 to find the polytope with vertices on the convex hull). The bigger the t-ratio, the more similar the data to the polytope. The t-ratio of the data was compared to that of 1,000 sets of shuffled data, produced by randomly permuting each coordinate of the data separately. p-value was defined as the fraction of sets for which the t-ratio is equal to or bigger than the t-ratio of the data.

To be stringent, the p-values reported in the paper are from the second method, as they were always larger than the p-values found by the first method.

### 1D gene enrichment at archetypes

To infer potential tasks of archetypes, we tested, for each gene, whether it is expressed maximally in the cells closest to one of the archetypes. To avoid circularity concerns, we removed that gene from the dataset, and recalculated the archetypes (in the D-1 dimensional space, where D is the number of genes). The changes in the archetype coordinates upon removal of the gene were minor (mean relative change 0.02%). We divided cells into bins according to their Euclidean distance from the archetype (computed in the D-1 gene expression space), and asked whether the expression of the removed gene is maximal in the bin closest to the archetype, and at what statistical significance. Choosing a number of bins between 5–25 left the results essentially unchanged (see S22 Fig). The p-value was computed using Wilcoxon rank-sum test [95], comparing the distribution of expression in the first bin (closest to archetype) to the distribution in other bins. We set a p-value threshold of p = 0.001, to avoid type II error concerns (multi-hypothesis testing). Shuffled data using the same tests resulted in 0.1 enriched genes on average, whereas the real data showed 70 enriched genes for the intestinal cells data.

### 2D gene enrichment at archetypes

In addition to the 1-dimensional leave-1-out enrichment check described above, we also performed a 2-dimenisonal enrichment check for selected marker genes. After taking out the selected gene, we projected the data on its 2 first PCA axes, and smoothed the marker values to obtain a 2D density function M(PC1,PC2). To smooth the data we used a Gaussian kernel function with variance computed by Silverman's Rule of Thumb [96], multiplied by a hill function of the density to avoid effects of low density of points (in low density regions the denominator is very small, resulting in artificial high marker values).

$$M\left(\overset{⃑}{x}\right)\;=\;\frac{\sum_{i\;=\;1}^{N}{m({\overset{⃑}{x}}_{i})e}^{-\frac{1}{2}\left(\overset{⃑}{x}-{\overset{⃑}{x}}_{i}\right)H\left(\overset{⃑}{x}-{\overset{⃑}{x}}_{i}\right)}}{\sum_{i\;=\;1}^{N}e^{-\frac{1}{2}\left(\overset{⃑}{x}-{\overset{⃑}{x}}_{i}\right)H\left(\overset{⃑}{x}-{\overset{⃑}{x}}_{i}\right)}}\;\frac{{\left(\sum_{i\;=\;1}^{N}e^{-\frac{1}{2}\left(\overset{⃑}{x}-{\overset{⃑}{x}}_{i}\right)H\left(\overset{⃑}{x}-{\overset{⃑}{x}}_{i}\right)}\right)}^{n}}{k^{n}+{\left(\sum_{i\;=\;1}^{N}e^{-\frac{1}{2}\left(\overset{⃑}{x}-{\overset{⃑}{x}}_{i}\right)H\left(\overset{⃑}{x}-{\overset{⃑}{x}}_{i}\right)}\right)}^{n}}$$

Where ${\overset{⃑}{x}}_{i}$ is the i^th^ data point, $m\left({\overset{⃑}{x}}_{i}\right)$ is the value of the marker at point ${\overset{⃑}{x}}_{i}$, H is a diagonal matrix $H\;=\;\left(\begin{array}{cc} h_{x} & 0\\ 0 & h_{y} \end{array}\right),\;h_{j}\;=\;{\left(\frac{4{\hat{\sigma_{j}}}^{5}}{3N}\right)}^{\frac{1}{5}}$, and k,n are the hill function coefficients, set to 8,2 respectively. We checked whether the location of the maxima of $M\left(\overset{⃑}{x}\right)$ is close to the computed position of the archetypes. This served as a qualitative check for enrichment.

### Downloadable software for Pareto task inference for biological datasets

A software package that fits polytopes to biological datasets, finds their significance, and computes features such as gene categories enriched near each archetype [33] can be found at http://wws.weizmann.ac.il/mcb/UriAlon/download/ParTI.

## Supporting Information

S1 Fig Removing up to 25% of the cells or the genes in the intestinal dataset has only a small effect on archetype positions. Relative error in archetypes is plotted as function of the fraction of cells (a) and genes (b) that were removed. The relative error was computed as the distance between the original archetypes position to the position of the archetypes that were computed after taking out the cells/genes with the lowest expression, normalized by the mean distance between all pairs of the original archetypes (which is a typical distance scale of the dataset).
(EPS)

S2 Fig Archetypal analysis is more robust to data sampling than clustering for the present dataset. Real data and two bootstrapping instances and their k-means clustering (color indicates cluster) show that cluster boundaries vary with resampling. For example, the red cell marked by a red arrow, is assigned to different clusters in different datasets created by bootstrapping (resampling of the data with replacement). In contrast, in archetypal analysis (bottom row, same bootstrapping instances as in top row) cells are defined by their distance from the archetypes (blue arrows), which is more robust to the sampling of the data, as quantified in S3 and S4 Figs.
(PDF)

S3 FigDescription of cells by archetypal analysis is more robust to data sampling than clustering or PCA.1,000 datasets were created by bootstrapping the data. Each dataset was clustered using k-means, UPGMA hierarchical clustering and self-organizing map with 2X2 grid, as well as analyzed by PCA and by archetypal analysis (AA). Each of the data points is a 76-dimensional gene expression vector for one cell $\vec{c}$. The different methods offer an approximated description $\tilde{\vec{c}}$ for each cell: a convex combination of the 4 archetypes (AA), a linear combination of the 3PCs (PCA), or the centroid of the cluster the cell is assigned to (clustering techniques). We computed the normalized standard deviation (SD) of the description of each cell $\tilde{\vec{c}}$ by each method over the bootstrapped datasets (S1C and S1J Text). (a) Percentage of cells with normalized standard deviation larger than 0.3 in archetypal analysis, the 3 clustering methods and PCA. (b) Cells are colored by the normalized SD of their descriptions by each of the methods (white = small SD, black = large SD). Cells in the middle of the data cloud show large variations in clustering assignment (dark colors).(EPS)

S4 Fig Distribution of standard deviation of archetypal analysis description of cells has a short tail compared to other methods. Histograms of the normalized SD upon bootstrapping, computed as described in S1C and S1J Text. Brown—archetypal analysis, yellow—k-means, light blue—hierarchical clustering (UPGMA), dark blue—self-organizing map (SOM), green—PCA. Archetypal analysis shows the distribution with the shortest tail, i.e. in AA description there are much fewer cells whose description is strongly dependent on the sampling of the data compared to the other methods.
(EPS)

S5 Fig Intestinal cells tetrahedron archetypes are enriched with known cell types marker genes. Examples of 1-dimensional and 2-dimensional enrichment plots show enrichment of marker genes near each intestinal archetype. (a) The goblet cell marker MUC2 [97] (b) The stem cell marker LGR5 [45] (c) The gene NODAL, a marker for the new group of Nodal cells described here. Enrichment was computed as described in Methods: 1D Gene enrichment at archetypes and Methods: 2D Gene enrichment at archetypes.
(EPS)

S6 Fig Axin2 levels, a proxy for the height of the cell in the crypt, reveal the developmental path of intestinal crypt cells. a. Axin2 levels in the intestinal cells tetrahedron, projected on the PC1-PC2 plane. b. Axin2 levels in cells taken only from the bottom of the crypt, projected on PC1-PC2 plain (PCA was performed on each group of cells separately). Grey color means value of zero (high in crypt).
(EPS)

S7 Fig Intestinal cells colored by Wanderlust pseudo-temporal order suggest differentiation direction from stem cells to mature cells. a. All cells projected on PC1-PC2 plain. b. progenitor cells projected on their PC1-PC2 plain. Inset: AXIN2 levels in progenitors cells. Grey points value is zero (i.e. high in crypt). Wanderlust is an algorithm for pseudo-temporal ordering [80].
(EPS)

S8 Fig Good statistical significance for a tetrahedron requires a few tens of points and improves rapidly with number of data points. Points were uniformly sampled from a unit tetrahedron, and p-value for a tetrahedron was computed based on these samples, as described in Methods: Statistical significance of best fit polytopes. Error bars represent standard error from 12 repeats.
(EPS)

S9 Fig Intestinal progenitor tetrahedron archetype gene profiles show expression of task-specific genes. Archetype gene expression profiles for intestinal progenitor cells measured by single-cell qPCR. Zero level represents the average expression of each gene in the dataset. Tetrahedron summarizes the results of AA and enrichment analysis which suggest 4 tasks corresponding to the archetypes: 1. Stemness, 2. Activate cell-type specific genes 3. Stop dividing, 4. Reduce global expression.
(EPS)

S10 Fig Mouse and human archetype gene profiles are highly correlated. Each data point represents the expression level of a gene from an archetype. There are 72 data points, 3 data points for each gene (one for each archetype in the triangle), that is 24 data points for each archetype. Correlation and p-value are shown. Enriched genes in archetypes are shown in Fig 5.
(EPS)

S11 Fig Cancer cells re-inhabit the Pareto front spanned by normal bottom crypt cells. a. Cancer cells (red) projected onto the space spanned by the first 2 PCs of normal cells (blue). The archetypes and enriched genes relate to the normal cells. The cells are the same cells which appear in Fig 5b (however Fig 5b compares mouse to human and therefore uses only a subset of the genes—those common to the mouse and human data). b. Variance explained by first k principal components suggests that effective data dimensionality is two. Green line: variance explained by PCA of shuffled data. Points represent mean values. Error bars, which represent 95% confidence intervals, are smaller than line width.
(EPS)

S12 Fig Single-cell mass cytometry data falls inside a 4D simplex with 5 vertices. (a) Variance explained by first k principal components suggests that effective data dimensionality is four. Green line: variance explained by PCA of shuffled data (Methods: Determining the number of archetypes). Points represent mean values. Error bars, which represent 95% confidence intervals, are smaller than line width. (b) Explained variance of best fit polytopes with k = 2–11 vertices suggests that quality of fit saturates at k = 5–6 vertices. Polytopes were found in the full 31D space using PCHA algorithm. (c) Projections of the best fit 4D simplex archetypes and the data on all the PC pairs (see Fig 6 for projection on the first 3 PCs).
(EPS)

S13 Fig Single-cell mass cytometry data archetypes are enriched with known cell type markers. Shown are enrichment curves for selected surface markers: CD45, CD3, CD19, CD20, CD4, CD8, and CD11b. Cells were divided into equal sized bins according to their distance from each archetype. Each marker expression was measured in these bins, resulting in 5 curves for each marker—one for each archetype: blue—archetype 1, orange—archetype 2, yellow—archetype 3, purple—archetype 4, green—archetype 5. For more details See S1G Text. For the complete list of enriched genes in each archetype see S5 Table.
(EPS)

S14 Fig Archetype gene expression profiles of bone marrow single-cell mass cytometry data indicate known cell types. Gene expression profiles of bone marrow single cell data acquired by single-cell mass cytometry [13,98]. Shown are log fold expression levels, zero level represents each gene’s average expression in the data set. S5 Table presents the list of enriched genes in every archetype.
(EPS)

S15 Fig Unidentified clusters in viSNE analysis of bone marrow cells correspond to cells found in the middle of the 4D simplex. viSNE analysis of the data carried by [13], colored by distance of cells from the archetypes in the 4d simplex (blue—closest cells to CD4 T-cells archetype; red—closest cells to monocytes archetype; yellow—closest cells to non-leukocyte archetype; purple—closest cells to CD8 T-cells archetype; green—closest cells to B cells archetype; gray—cells which are found in the middle of the simplex). Unidentified clusters in the viSNE analysis (not shown, see [13]) correspond to cells found in the middle of the tetrahedron, suggesting they are intermediates between known cell types.
(EPS)

S16 FigUniformity of the distribution of cell states varies between tissues.The similarity to a uniform distribution within the convex hull of the data was assessed for each data set. Data was projected on its first 3PCs. Local density *ρ*_*L*_ was computed as the mean density in a sphere of volume V around each datapoint, where V is the convex hull volume divided by the number of datapoints N. This process was repeated for a uniform distribution of points, yielding *ρ*_*U*_. Non-uniformity is defined as $=\;\frac{\rho_{L}}{\rho_{U}}$. Note that this definition controls for edge effects for points on the convex hull. A value of *ρ* = 1 is consistent with perfectly uniform data (green line), and higher values of *ρ* indicate more clumped or clustered data (see S1I Text). Mean values and error bars were calculated by sampling N uniformly distributed random points 10 times for each dataset.(EPS)

S17 Fig Archetypal analysis description of the data is more constrained than that of principal component analysis. Description by the 3 first PCs forces the data into a 3-dimensional space (left panel). Archetypal analysis description forces the data to be in a closed 3-d simplex, and hence is far more limiting (right panel). Thus, the fact that a 3D simplex (a tetrahedron) explains almost all of the variance that is explained by the first 3 PCs is remarkable.
(EPS)

S18 Fig Principal component vectors are not the same as the archetype profiles. Shown are the 3 PC vectors expression profiles (normalized to have norm 1). 0 represents each gene average value. Comparison to Fig 3 shows the differences from the archetype profiles. PC1 for example is composed roughly of archetype 3 (stem cells) minus archetype 1 (enterocytes), etc. No PC is dominated by the Nodal cells typical group of genes (true also for higher order PCs). For more details see S1J Text.
(EPS)

S19 Fig Principal component vectors are composed of a mixture of the archetypes. Archetype projections on the first 6 PCs. Heat map intensity represents a scalar product of archetypes and PCs. Note that PCs 1–3 are composed of a combination of the archetypes, and there is no one-to-one correlation between any pair of archetype and PC. PCs 4–6 do not contribute to further separation between the archetypes.
(EPS)

S20 Fig Increasing the number of archetypes may reveal more subtle trends. The archetypes split as additional archetypes are added to the analysis. The archetypes tree was generated by fitting the data to n archetypes and computing their Euclidean distance, in the 76-dimensional gene expression space, from the n-1 archetypes whose positions were computed before. Characterization of these archetypes was then done by carrying a leave-1-out enrichment analysis (Methods: 1D Gene enrichment at archetypes), and inspecting the enriched genes, shown in S7 Table.
(PDF)

S21 Fig Intestinal dataset shows bimodal technical error at low expression. (a) Scatter plot for gene with two primers (DLL4) shows high correlation between primers at high expression, and bimodal all/none type expression at low levels. (b) Probability that one primer shows zero expression as a function of expression of the other primer.
(EPS)

S22 Fig Number of enriched genes is weakly dependent on number of bins in the leave-one-out enrichment analysis. Analysis was performed on human intestinal dataset. Shown is the number of enriched genes at each archetype when carrying a leave-1-out enrichment analysis as described in Methods: 1D Gene enrichment at archetypes, using different bin sizes.
(EPS)

S23 Fig Explained variance curves suggest that dendritic cell RNA-Seq data is embedded in a 5D space. a. Explained variance of best fit polytopes with k = 2–11 vertices suggests that quality of fit saturates at k = 6 vertices. Polytopes were found in the full 500D space using PCHA algorithm. b. PCA eigenvalues loadings of real data (blue) vs. shuffled data (orange) suggest that the data can be approximated by a 5D space. Explained variance in both cases was computed as described in Methods: Determining the number of archetypes.
(EPS)

S24 Fig Single-cell RNA-Seq dendritic cell data archetypes have distinct gene expression profiles. Archetype gene expression profiles of dendritic cells stimulated with LPS, acquired by single-cell RNA-Seq [3]. Shown for each archetype are expression levels, normalized as described in Methods: Preprocessing and normalization of single-cell RNA-Seq data, of the 50 genes which deviate the most from their average expression level. Zero level represents each gene’s average expression in the data set. S6 Table presents the list of enriched functional gene groups in every archetype.
(EPS)

S1 Table Archetype positions are robust to the sampling of the data. Shown are errors in archetypes, computed based on 1,000 bootstrapped sets of the data. New set of archetypes was computed for each of these sets, resulting in 4 “clouds”, one around each archetype. The error is defined as the standard deviation divided by the distance of the center of the cloud from the origin. Shown are errors for the 3 first principal axes of noise for each of the archetypes (different axes for each archetype), and the mean error. (a) For the intestinal cells tetrahedron, (b) for intestinal progenitor cells tetrahedron. Visualization of these results can be seen in Figs 2c and 4b respectively.
(DOCX)

S2 Table Intestinal cells tetrahedron archetypes are enriched with specific sets of genes. Results of a leave-1-out enrichment analysis, carried as described in Methods: 1D enrichment at archetypes, using 10 bins and demanding p-value < 0.001 using Wilcoxon rank-sum statistical test.
(DOCX)

S3 Table When analyzed separately, mature intestinal cell types do not fall in a statistically-significant 2-4D polytope in gene expression space, in contrast to progenitor cells. Shown are p-values for each cell type to fall in a polytope with k vertices, k = 3–5. Enterocytes, goblet cells and nodal cells do not fall in a statistically significant polytope (all p-values>0.15), while progenitor cells fall in significant tetrahedron and triangle but not in a polytope with 5 vertices. P-values were computed as described in Methods: Statistical significance of best fit polytopes.
(DOCX)

S4 Table Intestinal progenitor cells archetypes are enriched with specific sets of genes. Results of a leave-1-out enrichment analysis, carried on intestinal progenitor cells tetrahedron as described in Methods: 1D enrichment at archetypes, using 5 bins and demanding p-value < 0.001 using Wilcoxon rank-sum statistical test.
(DOCX)

S5 Table Bone marrow cells leave-1-out enrichment reveals enriched genes at archetypes. Results of a leave-1-out enrichment analysis, carried on human bone marrow cells protein expression data, acquired by single-cell mass cytometry. Enrichment was computed as described in Methods: 1D enrichment at archetypes, using 10 bins and demanding p-value < 0.001 using Wilcoxon rank-sum statistical test.
(DOCX)

S6 Table Dendritic cells archetypes are enriched with robust specific functional gene groups. Functional gene groups (MSigDB gene sets [66]) enriched at archetypes and their significance levels. The functional categories that appear in this list were found to be robust to bootstrapping—they were enriched in the same archetype in 80% or more of 100 bootstrapped datasets (created by resampling with replacement of the original data, archetypes were computed again for each bootstrapped dataset). The p-value threshold to define significance was set using Benjamini-Hochberg test (FDR<0.1) to prevent multi-hypothesis testing error.
(XLSX)

S7 Table Enriched genes at archetypes were used to identify the splitting archetypes in S20 Fig. Leave-1-out enrichment results (Methods: 1D enrichment at archetypes, bin size = 0.1, demanding p-value lower than 0.001 using Rank-sum test) when looking for k = 2–6 archetypes for intestinal cells data. Archetypes titles in S20 Fig were given based on these results.
(XLSX)

S1 Text Extended methods and results. Estimation of technical noise and bimodality in the intestinal cell datasetRobustness of archetypes to the sampling of the data, intestinal datasetComparison of archetypal analysis to clustering methodsDefinition of cell types in the intestinal datasetEffect of sample size on the statistical significance of polytopesAnalysis of a single-cell qPCR dataset of a human colon cancer xenograft from a single cancer cellAnalysis of a single-cell mass cytometry dataset from human bone marrowAnalysis of single-cell RNA-Seq data for stimulated mouse spleen dendritic cellUniformity of the distribution of cell states varies between tissuesComparison of archetypal analysis to principal component analysisIncreasing the number of archetypes may reveal subtle trends
(PDF)

S1 Dataset Human intestinal cells gene expression obtained by single-cell qPCR, from Dalerba et al 2011, after processing and normalization as described in Methods: Preprocessing and normalization of single-cell qPCR data. (CSV)

S2 Dataset Human intestinal cell progenitors gene expression obtained by single-cell qPCR, from Dalerba et al 2011, after processing and normalization as described in Methods: Preprocessing and normalization of single-cell qPCR data. The cells analyzed here are a subset of the cells presented in S1 Dataset, chosen as described in S1D Text.
(CSV)

S3 Dataset Mouse intestinal cells gene expression obtained by single-cell qPCR, from Rothenberg et al 2012, after processing and normalization as described in Methods: Preprocessing and normalization of single-cell qPCR data. (CSV)

S4 Dataset Human cancer xenograft in mouse gene expression obtained by single-cell qPCR, from Dalerba et al 2011, after processing and normalization as described in Methods: Preprocessing and normalization of single-cell qPCR data, S1F Text. (CSV)

S5 Dataset Human bone marrow gene expression obtained by single-cell mass cytometry, from Bendall et al 2011, Amir et al 2013, after processing and normalization as described in Methods: Preprocessing and normalization of single-cell mass cytometry data, S1G Text. (CSV)

S6 Dataset LPS stimulated dendritic cells gene expression obtained by single-cell RNA-Seq, from Jaitin et al 2014, after processing and normalization as described in Methods: Preprocessing and normalization of single-cell RNA-Seq data, S1H Text. (CSV)

S7 Dataset Human intestinal cells gene expression obtained by single-cell qPCR, from Dalerba et al 2011, no further processing and normalization. This dataset includes 34 genes not included in the original publication, kindly provided by Dalerba et al.
(XLSX)
