# Supplementary figures and images for: Geometry of the Gene Expression Space of Individual Cells
Source: PLoS Comput Biol. 2015 Jul 10;11(7):e1004224. doi: 10.1371/journal.pcbi.1004224 (PMC4498931; doi:10.1371/journal.pcbi.1004224)

Real data

Bootstrapping1

Bootstrapping2

K - means

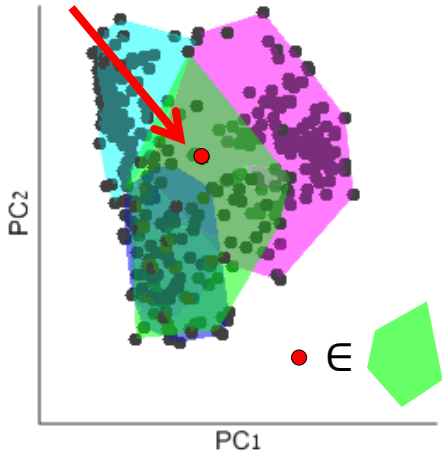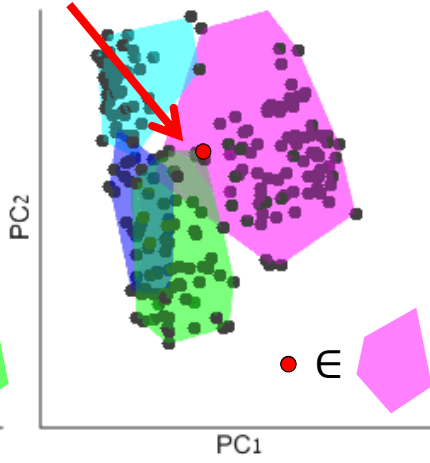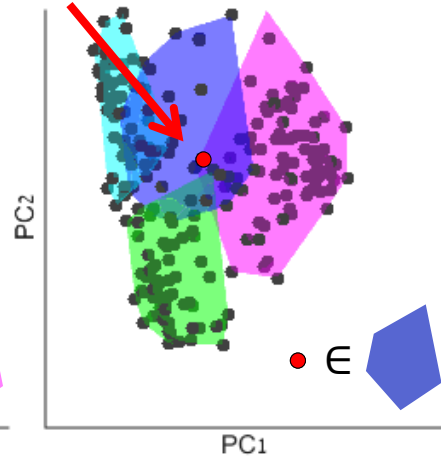

Archetypal analysis

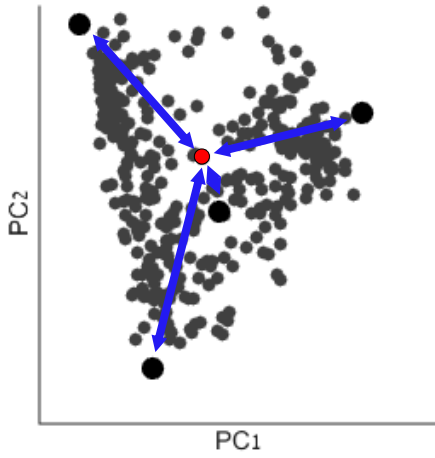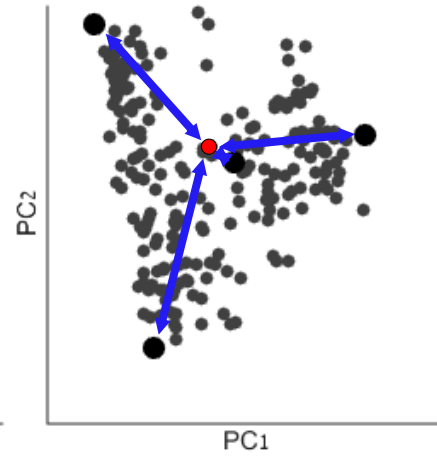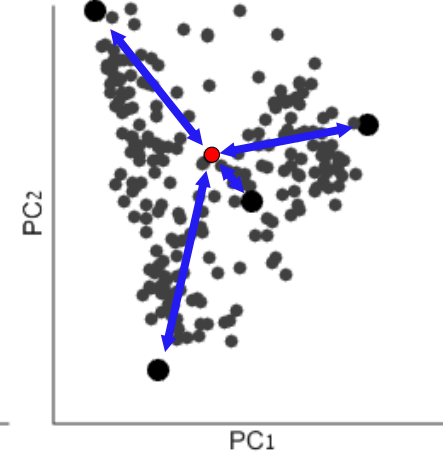

Supplement: S2 Fig — Real data and two bootstrapping instances and their k-means clustering (color indicates cluster) show that cluster boundaries vary with resampling. For example, the red cell marked by a red arrow, is assigned to different clusters in different datasets created by bootstrapping (resampling of the data with replacement). In contrast, in archetypal analysis (bottom row, same bootstrapping instances as in top row) cells are defined by their distance from the archetypes (blue arrows), which is more robust to the sampling of the data, as quantified in S3 and S4 Figs. (PDF) [file pcbi.1004224.s002.pdf]

Number of archetypes

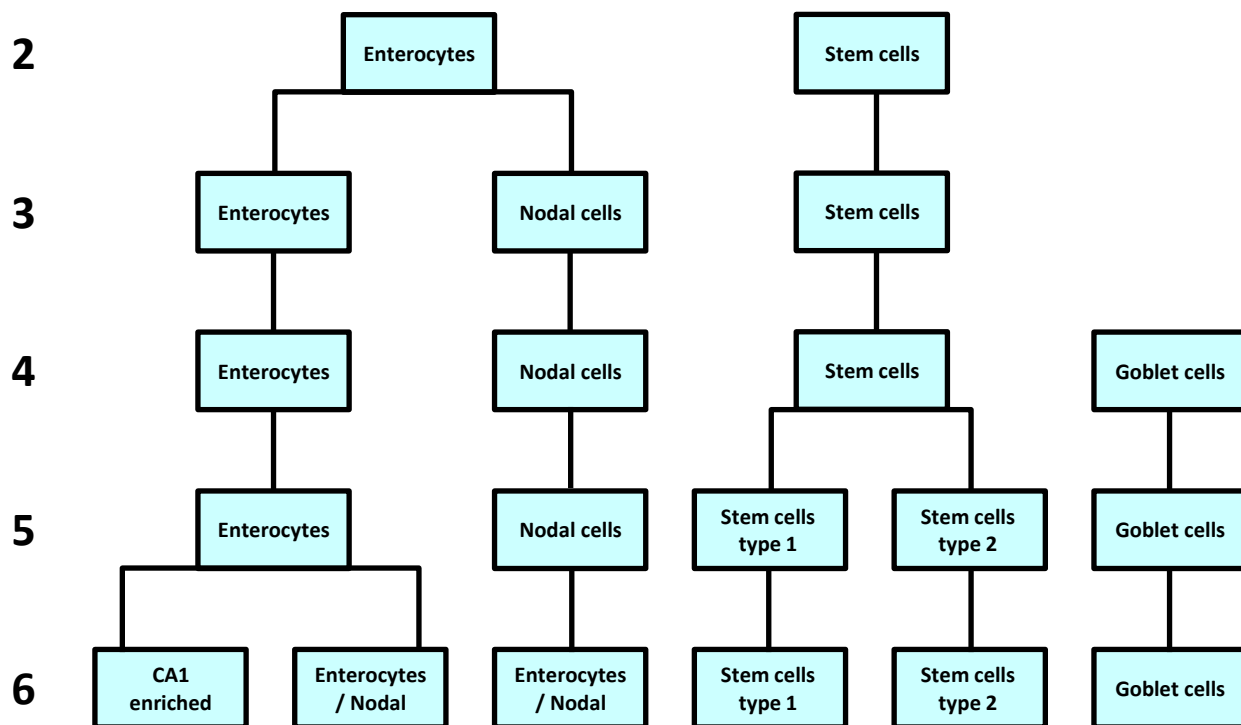

Supplement: S20 Fig — The archetypes split as additional archetypes are added to the analysis. The archetypes tree was generated by fitting the data to n archetypes and computing their Euclidean distance, in the 76-dimensional gene expression space, from the n-1 archetypes whose positions were computed before. Characterization of these archetypes was then done by carrying a leave-1-out enrichment analysis (Methods: 1D Gene enrichment at archetypes), and inspecting the enriched genes, shown in S7 Table. (PDF) [file pcbi.1004224.s020.pdf]
