# Supplementary material for: Geometry of the Gene Expression Space of Individual Cells
Source: PLoS Comput Biol. 2015 Jul 10;11(7):e1004224. doi: 10.1371/journal.pcbi.1004224 (PMC4498931; doi:10.1371/journal.pcbi.1004224)
Supplement: S1 Table — Shown are errors in archetypes, computed based on 1,000 bootstrapped sets of the data. New set of archetypes was computed for each of these sets, resulting in 4 “clouds”, one around each archetype. The error is defined as the standard deviation divided by the distance of the center of the cloud from the origin. Shown are errors for the 3 first principal axes of noise for each of the archetypes (different axes for each archetype), and the mean error. (a) For the intestinal cells tetrahedron, (b) for intestinal progenitor cells tetrahedron. Visualization of these results can be seen in Figs 2c and 4b respectively. (DOCX) [file pcbi.1004224.s025.docx]

**Table S1: Archetype positions are robust to the sampling of the data.** Shown are errors in archetypes, computed based on 1,000 bootstrapped sets of the data. New set of archetypes was computed for each of these sets, resulting in 4 “clouds”, one around each archetype. The error is defined as the standard deviation divided by the distance of the center of the cloud from the origin. Shown are errors for the 3 first principal axes of noise for each of the archetypes (different axes for each archetype), and the mean error. (a) For the intestinal cells tetrahedron, (b) for intestinal progenitor cells tetrahedron. Visualization of these results can be seen in Fig. 2c and Fig. 4b respectively.

**a.**

|  | **Arc 1 Enterocytes (%)** | **Arc 2 Nodals (%)** | **Arc 3 Stem cells**  **(%)** | **Arc 4 Goblet cells**  **(%)** |
| --- | --- | --- | --- | --- |
| 1^st^ noise principal axis | 10.6 | 14 | 5.9 | 19.9 |
| 2^nd^ noise principal axis | 6.2 | 7.4 | 3.7 | 9.8 |
| 3^rd^ noise principal axis | 1.8 | 5.1 | 1.8 | 3.1 |
| **Average error** | **6.2** | **8.8** | **3.8** | **10.9** |

**b.**

|  | **Arc 1 (%)** | **Arc 2 (%)** | **Arc 3 (%)** | **Arc 4 (%)** |
| --- | --- | --- | --- | --- |
| 1^st^ noise principal axis | 14.2 | 18.8 | 12.1 | 11.4 |
| 2^nd^ noise principal axis | 6.1 | 6.4 | 6.5 | 6.3 |
| 3^rd^ noise principal axis | 4.7 | 4.1 | 5.5 | 2.9 |
| **Average error** | **8.3** | **9.8** | **8** | **6.9** |
