# Supplementary material for: Geometry of the Gene Expression Space of Individual Cells
Source: PLoS Comput Biol. 2015 Jul 10;11(7):e1004224. doi: 10.1371/journal.pcbi.1004224 (PMC4498931; doi:10.1371/journal.pcbi.1004224)
Supplement: S2 Table — Results of a leave-1-out enrichment analysis, carried as described in Methods: 1D enrichment at archetypes, using 10 bins and demanding p-value < 0.001 using Wilcoxon rank-sum statistical test. (DOCX) [file pcbi.1004224.s026.docx]

**Table S2: Intestinal cells tetrahedron archetypes are enriched with specific sets of genes.** Results of a leave-1-out enrichment analysis, carried as described in Methods: 1D enrichment at archetypes, using 10 bins and demanding p-value $<$ 0.001 using Wilcoxon rank-sum statistical test.

| **Arc 1 (Enterocytes)** | **Arc 2 (Nodals)** | **Arc 3 (Stem cells)** | **Arc 4 (Goblet cells)** |
| --- | --- | --- | --- |
| AQP8 | CFC1 | AGR2 | AGR2 |
| CD177 | CLDN8 | AQP1 | CDH1 |
| CEACAM1 | NODAL | ASCL2 | CDKN1A |
| KRT20 | PCGF6 | AXIN2 | DLL1 |
| MS4A12 | TDGF1 | BMI1 | DLL4 |
| SLC26A3 | UGT1A1 | CDCA7 | EPCAM |
|  |  | CDH1 | MLLT10 |
|  |  | CFTR | MUC2 |
|  |  | CLDN7 | SLC12A2 |
|  |  | DLL4 | SPDEF |
|  |  | ECT2 | SPINK4 |
|  |  | EGFR | TCF7L2 |
|  |  | EPCAM | TERT |
|  |  | FERMT1 | TFF3 |
|  |  | FSCN1 | USP16 |
|  |  | GAPDH |  |
|  |  | HES1 |  |
|  |  | KIF12 |  |
|  |  | LEFTY1 |  |
|  |  | LGR5 |  |
|  |  | LRIG1 |  |
|  |  | METTL3 |  |
|  |  | MYC |  |
|  |  | NAMPT |  |
|  |  | NOTCH1 |  |
|  |  | OLFM4 |  |
|  |  | PTPLAD1 |  |
|  |  | PTPRO |  |
|  |  | RGMB |  |
|  |  | RNF43 |  |
|  |  | SEC62 |  |
|  |  | SLC12A2 |  |
|  |  | STMN1 |  |
|  |  | TSPAN6 |  |
|  |  | UGT2B17 |  |
|  |  | UGT8 |  |
