# Supplementary material for: Geometry of the Gene Expression Space of Individual Cells
Source: PLoS Comput Biol. 2015 Jul 10;11(7):e1004224. doi: 10.1371/journal.pcbi.1004224 (PMC4498931; doi:10.1371/journal.pcbi.1004224)
Supplement: S3 Table — Shown are p-values for each cell type to fall in a polytope with k vertices, k = 3–5. Enterocytes, goblet cells and nodal cells do not fall in a statistically significant polytope (all p-values>0.15), while progenitor cells fall in significant tetrahedron and triangle but not in a polytope with 5 vertices. P-values were computed as described in Methods: Statistical significance of best fit polytopes. (DOCX) [file pcbi.1004224.s027.docx]

**Table S3: When analyzed separately, mature intestinal cell types do not fall in a statistically-significant 2-4D polytope in gene expression space, in contrast to progenitor cells.** Shown are p-values for each cell type to fall in a polytope with k vertices, k=3-5. Enterocytes, goblet cells and nodal cells do not fall in a statistically significant polytope (all p-values>0.15), while progenitor cells fall in significant tetrahedron and triangle but not in a polytope with 5 vertices. P-values were computed as described in Methods: Statistical significance of best fit polytopes.

| **#archetypes** | **progenitors** | **enterocytes** | **goblet cells** | **NODAL** |
| --- | --- | --- | --- | --- |
| 3 | 0.02 | 0.18 | 0.84 | 0.33 |
| 4 | 0.01 | 0.55 | 0.16 | 0.34 |
| 5 | 0.17 | 0.78 | 0.21 | 0.48 |
