# Supplementary material for: Geometry of the Gene Expression Space of Individual Cells
Source: PLoS Comput Biol. 2015 Jul 10;11(7):e1004224. doi: 10.1371/journal.pcbi.1004224 (PMC4498931; doi:10.1371/journal.pcbi.1004224)
Supplement: S4 Table — Results of a leave-1-out enrichment analysis, carried on intestinal progenitor cells tetrahedron as described in Methods: 1D enrichment at archetypes, using 5 bins and demanding p-value < 0.001 using Wilcoxon rank-sum statistical test. (DOCX) [file pcbi.1004224.s028.docx]

**Table S4: Intestinal progenitor cells archetypes are enriched with specific sets of genes.** Results of a leave-1-out enrichment analysis, carried on intestinal progenitor cells tetrahedron as described in Methods: 1D enrichment at archetypes, using 5 bins and demanding p-value $<$ 0.001 using Wilcoxon rank-sum statistical test.

| **Arc1** | **Arc2** | **Arc3** | **Arc4** |
| --- | --- | --- | --- |
| AQP1 | CA1 | ACTB |  |
| ASCL2 | CA2 | CDH1 |  |
| AXIN2 | CD177 | CDKN1A |  |
| CDCA7 | CFTR | CLDN1 |  |
| CDK6 | DPP4 | KRT20 |  |
| FERMT1 | SLC26A3 | PHLDA1 |  |
| LEFTY1 | SPDEF | TCF7L2 |  |
| LGR5 | UGT2B17 | USP16 |  |
| METTL3 |  |  |  |
| PTPRO |  |  |  |
| RGMB |  |  |  |
| RNF43 |  |  |  |
| SLC12A2 |  |  |  |
| TERT |  |  |  |
| TFF3 |  |  |  |
