# Supplementary material for: Geometry of the Gene Expression Space of Individual Cells
Source: PLoS Comput Biol. 2015 Jul 10;11(7):e1004224. doi: 10.1371/journal.pcbi.1004224 (PMC4498931; doi:10.1371/journal.pcbi.1004224)
Supplement: S5 Table — Results of a leave-1-out enrichment analysis, carried on human bone marrow cells protein expression data, acquired by single-cell mass cytometry. Enrichment was computed as described in Methods: 1D enrichment at archetypes, using 10 bins and demanding p-value < 0.001 using Wilcoxon rank-sum statistical test. (DOCX) [file pcbi.1004224.s029.docx]

**Table S5: Bone marrow cells leave-1-out enrichment reveals enriched genes at archetypes.** Results of a leave-1-out enrichment analysis, carried on human bone marrow cells protein expression data, acquired by single-cell mass cytometry. Enrichment was computed as described in Methods: 1D enrichment at archetypes, using 10 bins and demanding p-value $<$ 0.001 using Wilcoxon rank-sum statistical test.

| **Archetype 1** | **Archetype 2** | **Archetype 3** | **Archetype 4** | **Archetype 5** |
| --- | --- | --- | --- | --- |
| CD3 | CD45 | CD123 | Btk/Itk | Ki67 |
| CD4 | CD45RA | CD19 | CD11b |  |
| CD45 | CD8 | CD20 | CD123 |  |
| IkBalpha | IkBalpha | CD34 | CD33 |  |
|  |  | CD45 | CD38 |  |
|  |  | CD45RA | CD4 |  |
|  |  | CD90 | CD45 |  |
|  |  | ERK1/2 | CD90 |  |
|  |  | P38 | CREB |  |
|  |  | PLCgamma2 | CrkL |  |
|  |  | S6 | ERK1/2 |  |
|  |  | SHP2 | H3 |  |
|  |  | SLP-76 | IkBalpha |  |
|  |  | STAT3 | MAPKAPK2 |  |
|  |  | ZAP70/Syk | NFkB |  |
|  |  |  | P38 |  |
|  |  |  | PLCgamma2 |  |
|  |  |  | S6 |  |
|  |  |  | SHP2 |  |
|  |  |  | SLP-76 |  |
|  |  |  | SrcFK |  |
|  |  |  | STAT3 |  |
|  |  |  | STAT5 |  |
|  |  |  | ZAP70/Syk |  |
