# Supplementary material for: Geometry of the Gene Expression Space of Individual Cells
Source: PLoS Comput Biol. 2015 Jul 10;11(7):e1004224. doi: 10.1371/journal.pcbi.1004224 (PMC4498931; doi:10.1371/journal.pcbi.1004224)
Supplement: S1 Text — Estimation of technical noise and bimodality in the intestinal cell datasetRobustness of archetypes to the sampling of the data, intestinal datasetComparison of archetypal analysis to clustering methodsDefinition of cell types in the intestinal datasetEffect of sample size on the statistical significance of polytopesAnalysis of a single-cell qPCR dataset of a human colon cancer xenograft from a single cancer cellAnalysis of a single-cell mass cytometry dataset from human bone marrowAnalysis of single-cell RNA-Seq data for stimulated mouse spleen dendritic cellUniformity of the distribution of cell states varies between tissuesComparison of archetypal analysis to principal component analysisIncreasing the number of archetypes may reveal subtle trends (PDF) [file pcbi.1004224.s032.pdf]

Supplementary Information

Contents

Supplementary Text 2

A. Estimation of technical noise and bimodality in the intestinal cell dataset 2

B. Robustness of archetypes to the sampling of the data, Intestinal dataset 2

C. Comparison of archetypal analysis to clustering methods 3

D. Definition of cell types in the Intestinal dataset 4

E. Effect of sample size on the statistical significance of polytopes 5

F. Analysis of a single-cell qPCR dataset of a human colon cancer xenograft from a single cancer cell 5

G. Analysis of a single-cell mass cytometry dataset from human bone marrow 6

H. Analysis of a single-cell RNA-Seq dataset of stimulated mouse spleen dendritic cell ... 7

I. Uniformity of the distribution of cell states varies between tissues 9

J. Comparison of archetypal analysis to Principal Component Analysis 10

K. Increasing the number of archetypes may reveal subtle trends 11

References 12

Supplementary Text

A. Estimation of technical noise and bimodality in the intestinal cell dataset

In the data of [1], 5 of the genes had duplicate primers, allowing estimation of technical noise and bimodality effects. For all 5 genes, measures were highly correlated at high expression, but for low expression, often one of the primers displayed a read of 0, leading to a bimodal effect as described in [2,3]. Since bimodality occurs only at low expression levels (S21a Figure), we assumed that they are due to failure of one primer to sample the gene. Thus, for the 5 genes that had duplicate primers, we took the mean expression of the primers, unless one of them was zero – in which case we took the non-zero value. See S21b Figure for analysis of false-negative error (i.e. the chance of a gene to be detected only by one of the primers) for different expression levels. Since the error is highest at low expression, we removed 10% genes and cells with low expression as described in methods. We compared this to the method of McDavid et al [2] for removing outlier cells, and find excellent agreement (89% overlap in the cells removed by the two methods, using parameters which remove 10% of the cells).

B. Robustness of archetypes to the sampling of the data, Intestinal dataset

In order to test the robustness of the method to the specific sampling of the data, we created 1,000 randomized datasets by bootstrapping the intestinal dataset (resampling the data with replacements to reach the same size as the original dataset). We computed the positions of the archetypes for each of the datasets. Table S1 shows the errors in each of the archetypes. The relative errors were calculated by computing the variance in each of the 3 principal axes of the noise (variation between bootstrapped datasets) in each archetype, taking their mean value, and dividing by the distance of the archetype from the origin (the dataset mean). Figure 2c-f and 4b show the archetype positions mean and standard deviation, represented by ellipsoids whose principal axes are aligned with the principal axes of the noise in each archetype, and their length is the standard deviation of the archetype position in these directions.

We also tested the robustness of the list of enriched genes in every archetype under bootstrapping, using the same 1D enrichment method as for the original dataset. All of the enriched genes in the list repeated in at least 50% of the bootstrapped data sets, except for 6 genes that were consequently removed. The inferred tasks remained the same under bootstrapping.

C. Comparison of archetypal analysis to clustering methods

When studying the composition of a tissue, several clustering techniques can be used to classify cells into subtypes based on their gene expression [4]. These methods give a discrete description of the data, by assigning each cell to one group:

$$\vec{x}_{est} = \sum_{i=1}^{N} \theta_i \vec{v}_i \ , \qquad \sum_{i=1}^{N} \theta_i = 1, \qquad \theta_i = 0 \ or \ 1$$

Where $\vec{x}_{est}$ is the approximated description of data point (cell) $\vec{x}$, $\vec{v}_i$ are the clusters centroids, and N is the number of clusters.

The description suggested by archetypal analysis is different because it is continuous: Each cell is represented by its position inside the polytope, given by its distance from the archetypes:

$$\vec{x}_{est} = \sum_{i=1}^{N} \theta_i \vec{a}_i \ , \qquad \sum_{i=1}^{N} \theta_i = 1, \qquad \theta_i \in [0\ ,1]$$

Where $\vec{a}_i$ are the archetypes vectors, and N is the number of archetypes.

The Pareto-inspired interpretation of the polytopal geometry [5] of the data further suggests a biological insight in terms of the tasks of the system and their tradeoffs, which is lacking in clustering approaches.

In this section we compare the sensitivity of the description of the data by archetypal analysis to the sensitivity of its description by 3 commonly used clustering methods: k-means clustering [6], UPGMA hierarchical clustering [7], and self-organizing maps [8,9].

In order to check the sensitivity of the different methods to the sampling of the data, we created 1,000 random datasets by bootstrapping (resampling of the data points with replacement). For each bootstrapped dataset we found archetypes using the PCHA algorithm [10] and clustered the data using the three methods.

Each cell in each dataset was described by 76 coordinates. In archetypal analysis – the coordinates of the best description of the cell by linear combination of the archetypes:

$\vec{x}_{est} = \sum_{i=1}^{N} \theta_i \vec{a}_i,$

and in clustering – the centroid of the cluster that the cell is assigned to:

$\vec{x}_{est} = \sum_{i=1}^{N} \theta_i \vec{v}_i$

We gathered these descriptions for each cell, for each of the methods. Then we computed the normalized standard deviation (SD) for each cell by dividing the standard deviation along the 1st principal component of variation in these descriptions by the standard deviation of the 1st principal component of the complete real data:

$$\sigma_{norm} = \frac{\sigma(1^{st}\ PC\ of\ \vec{x}_{est})}{\sigma(1^{st}\ PC\ of\ data)}$$

Visualization of the results can be seen in Figure S3b, where cells are colored by their normalized SD in each of the methods and in Figure S4 which shows the distribution of the normalized SD among cells. Note that cells next to the vertices of the polygon have low SD in all of the methods (in UPGMA even lower than in archetypal analysis), since their assignment to a cluster is robust. However, cells in the middle of the dataset, which do not belong naturally to any particular cluster, have high SD in all of the three clustering methods. These cells are assigned in different bootstrapped datasets to different clusters, since the boundaries of the clusters are sensitive to the specific sampling of the data (S2 Figure top row). In contrast, the description of these cells by archetypal analysis is robust to the sampling of the data, since the archetype positions are robust as well as the cell's distance from the archetypes (S2 Figure bottom row).

Mathematically, $\vec{v}_i$ and $\vec{a}_i$ are both fairly robust to the sampling of the data (see Table S1), but because of the different constrains, $\vec{\theta}$ in clustering is not robust to sampling while in archetypal analysis it is, in data with a geometry such as the present dataset. Note that if the data was arranged in distinct, well-separated clouds, both clustering and AA would be robust to sampling.

The robustness of archetype analysis to data sampling in comparison to the other methods tested here is summarized in S3a Figure, that shows the percentage of cells with high normalized SD (variation between bootstrapping datasets larger than 0.3 of the variation captured by the first PC of the real dataset), which is less than 1% in archetypal analysis and more than 14% in all of the other methods.

D. Definition of cell types in the Intestinal dataset

We examined two methods to divide the data points into cell types.

First, we used manual clustering that was done in [1] according to marker genes CA1, SLC26A3 (enterocytes), LGR5, ASCL2, OLFM4, CA2 (progenitors), MUC2 ,TFF3 (goblet cells) and NODAL.

Second, we assigned each data point to a cell type according to the closest archetype (for example, if a point was closer to the enterocyte archetype than to other archetypes, it was marked as an enterocyte). Sorting by the two methods gave similar results (85% overlap).

This second method classifies the Nodal cells as well, which were not previously recognized. Note that the published data in Dalerba et al does not include 34 genes, included in the present analysis, kindly provided by Dalerba et al for this study. A subset of these genes allows identification of the Nodal class.

Finally, we also used t-SNE, an algorithm for non-linear projection of the data that conserves the pairwise distance between points [11]. We projected the data into 2D using t-SNE, and then used k-means clustering with distance measure of one minus the cosine of the included angle between points, and 20 replicates starting from arbitrary initial cluster centroids, to divide the data into 4 clusters. Again, classification agreed with the other methods (79% overlap).

E. Effect of sample size on the statistical significance of polytopes

Practical use of the present approach raises the question of how much data is needed to discover polytopes reliably. To test this, in this section we study the effect of sample size needed to attain statistical significance for tetrahedral geometry, given that the data lies perfectly in a tetrahedron. We randomly sampled points from a perfect tetrahedron and checked the p-value for a tetrahedron by applying our algorithm to this data. The test was done for 20 to 70 points, and the p-value was computed as described in Methods: Statistical significance of best fit polytopes. Results are shown in S8 Figure. 20 points yield a non-significant p-value of 0.2; 30 points are enough to get a p-value of 0.04; while for 40 points the p-value is already small – 0.003. In general, the p-value appears to drop exponentially with the number of points. However, considering technical and biological noise as well as other effects beside trade-offs that may be in play, one should expect higher p-values when considering real datasets.

In light of these results, it is not surprising that when testing each cell type separately goblet cells (18 cells) do not show a significant polytope. However, enterocytes (89 cells) and nodal cells (114 cells) could in theory yield a significant p-value. The fact that they do not show such geometry may suggest that the current approach may not be useful for these cells, since other effects dominate the structure of the data. Alternatively, tradeoff may exist in these cells, but is not manifested in the set of genes that were measured in this study. These cells might also have too many competing tasks (too many archetypes) to be detectable.

F. Analysis of a single-cell qPCR dataset of a human colon cancer xenograft from a single cancer cell

An important question that can now be studied using single-cell technologies is the heterogeneity of cancer tumors, and specifically, whether tumors heterogeneity recapitulates developmental processes in normal tissues [1,12–15]. We studied a dataset of human colon cancer xenograft, created from a single human cancer cell that was injected to mice, as described in [1], which contained the expression of 90 genes in 589 cells.

We pre-processed the data as described in *Methods: Processing and normalization of the data*. We united 5 genes with multiple primers as described in S1 Text Section A, and removed low expressed genes and low expressing cells, to obtain a dataset of 521 cells and 76 genes.

We found that the cancer cells data is, to a good approximation, low dimensional – only 2 PCs explained 37% of the variance in the data, while the third PC explained only 4% more (S11b Figure). In order to compare between the cancer data and the normal tissue data, we projected the cancer cells into the space spanned by the 2 first PCs of the healthy bottom crypt cells (the two datasets included the same genes). The normal cells are the cells that appear in Fig. 5b (though in Fig. 5b the analysis was carried using only a subset of genes to compare with mouse data (Fig. 5a)).

In agreement with Dalerba et al [1], we notice that the cancer cells re-inhabit the Pareto front spanned by the normal cells, with a similar density distribution (compare Fig. 5(b), Fig. S11(a)). This hints that the human cancer cells undergo differentiation similar to the normal mouse and human tissues.

G. Analysis of a single-cell mass cytometry dataset from human bone marrow

We analyzed data of protein levels in human healthy bone marrow cells, acquired by single-cell mass cytometry [11,16]. The data contained measurements of 31 protein levels in 10,000 cells, and was preprocessed and normalized as described in Methods.

First, we asked whether the data geometry is polyhedral. We found that the data is approximately 4 dimensional and that it is well described by a 4D polytope with 5 vertices (p-value 0.005, Fig. 6, S12 Figure (a) and (b)). A 4D simplex explains 52% of the variance in the data, giving a description almost as accurate as the full 4D space spanned by the first 4 PCs, which explains 54%. Projections of the simplex on all the PC pairs are shown in S12(c) Figure. .

Next, we identified the archetypes and inferred the tasks they perform. In order to do so we examined the archetypes gene expression profiles (S14 Figure) and carried a leave-1-out enrichment analysis (S13 Figure, Table S5, Methods: 1D Gene enrichment at archetypes). We found that 4 of the archetypes showed marker genes identified with a known cell type. Archetype 1 is enriched with surface markers CD3 and CD4, and thus represents CD4 T cells. Archetype 2 is enriched with CD8 and express high CD33 and thus recognized with CD8 T-cells; Archetype 3 is enriched with CD19 and CD20, markers of B-cells; And archetype 4 is enriched with CD11b and CD33, markers of macrophages/ monocytes. All of these archetypes are enriched with CD45, a marker of leukocytes. The last archetype does not express this marker, and is not enriched for any other gene except from division marker ki67. We hypothesize this archetype represents non-leukocytes, which express proteins that were not included in this dataset.

One unexpected feature of this dataset is the existence of cells in the middle of the simplex. This may correspond to cells in the original viSNE analysis [11] that were not amenable to classification (S15 Figure). Based on the present results, one may hypothesize that some bone marrow cells express a combination of markers which does not label them as classic cell types.

H. Analysis of a single-cell RNA-Seq dataset of stimulated mouse spleen dendritic cell

Dendritic cells are known to carry out a wide range of immune functions, such as pathogen recognition, antigen presentation and regulation of lymphocytes [17–19]. It has been suggested that these functions cannot all be carried out by the same cell, and evidence for the existence of DC subtypes was found [20–24]. However, these cells cannot be easily clustered based on their gene expression profiles [25]. We therefore applied the Pareto approach to analyze dendritic cells, hypothesizing that they may reveal tradeoffs between immune tasks.

We used the single-cell RNA-Seq data from [25], of mice spleen CD11c+ cells. Each cell was characterized by 20,091 gene expression counts, based on sampling a fraction of the cell's mRNA pool. This data was classified by Jaitin et al into seven groups using a probabilistic mixture model. One group of cells, however, seemed to defy clear clustering (class VII in [25]) – These are the dendritic cells in the spleen. We therefore analyzed LPS stimulated dendritic cells. We performed down-sampling as in [25] and focused on the 500 genes with the highest standard deviation across samples, resulting in 312 cells with 400 mRNA counts per cell. Results were similar for choosing genes by highest mean or median. The data was preprocessed and normalized as described in Methods.

We find that the data is well described by a tetrahedron (p<10^-3, Fig. 6 bottom row). A tetrahedron explains about 10% of the variance in the data (S23a Figure). This fraction of explained variance is lower than in the other datasets analyzed in this study, presumably because of the large number of genes in the RNA-Seq dataset (500 versus a few tens in the qPCR and cytof methods) which may contribute larger experimental variability. When inspecting the eigenvalue loadings of the data compared to shuffled data (see Methods: determining the number of archetypes, S23b Figure), it seems that the data is embedded in a 5 dimensional space. However, we chose to describe the data by a 3D polytope since the relatively small number of cells and of reads per cell does not allow finding robust archetypes in 5D space (5D archetypes are not stable under bootstrapping). It is thus possible that some of the archetypes that we find may represent unification of higher order archetypes.

To study the archetypes tasks, we carried an enrichment analysis on biological functions (MSigDB gene sets [26]), using parts of ParTI software package [27]. In this analysis we considered all 10,208 genes that were expressed in the LPS-stimulated dendritic cells. The value for each gene group in each cell was set to be the average value of the expression of genes that belong to this group. We binned the cells according to their Euclidean distance from each archetype (in the 500D gene expression space) and asked which biological functions are enriched maximally in the cells closest to each archetype, as described in *Methods: 1D enrichment analysis*.

To address concerns about multiple hypothesis testing [28,29], we used the Benjamini-Hochberg (FDR<0.01) test to ensure that the functional categories found significant are not due

to the fact that we test many categories. As a second test, we shuffled the data and repeated the enrichment analysis, to find that only 34 ± 14 functional categories are enriched on average in shuffled data compare to 1164 in the real data (total over all archetypes). We conclude that type-II errors do not explain the present enrichment.

To remove concerns about sensitivity to sampling, raised because of the sparseness of the data, we further checked the sensitivity of the enrichment to bootstrapping of the data. We resampled the data with replacement 100 times and inspected only gene groups that were statistically significantly enriched in the same archetype in more than 80% of the bootstrapped data sets (see Table S6 for the full list, S24 Figure shows the archetypes gene expression profiles).

The results suggest that dendritic cells trade-off between 4 key immune tasks: (i) response to virus (cytoplasmic DNA response and interferon pathways) (ii) Dendritic cell maturation, and formation of cytoskeletal features. (iii) Antigen presentation and activation of lymphocytes (iv) putative apoptosis pathways.

The first archetype is enriched with anti-viral functions and interferon pathways. It shows high expression of genes such as Stat2, Irf7, Ifit3 and Ifit1 that were previously described as part of an anti-viral module which is expressed only in a subset of dendritic cells [18,22]. Cells closest to this archetype correspond to this previously described group.

The second archetype is enriched with cytoskeleton organization and biogenesis functions. It is enriched with genes Tmem176a and Tmem176b, that were previously reported as marking an immature dendritic cell state [30]. In addition it expresses Ccr7, which is present in migrating cells [24,31]]. We hypothesize that this archetype represents cells that are found in a maturation process. A main task of these cells is phagocytosis, which also requires high cytoskeletal activity [32–34]. Enrichment of GO terms such as lytic vacuole, vacuole and lysosome strengthen this hypothesis.

The third archetype is enriched with cytokine production and secretion, and cell-cell signaling. Highly expressed genes in this archetypes are chemokines including Ccl5, Ccl22, Ccl4, and antigen presentation related genes such as subunit IL27, H2-Eb1, H2-Aa, and Cd74. Cells near this archetype are likely to be active mature dendritic cells [22,35,36]. Their task is antigen presentation and stimulation of lymphocytes including T cells.

The fourth archetype is harder to interpret. It is enriched with, apoptosis pathways, activity in mitochondria and membrane parts, and sphingolipids metabolism. Therefore we hypothesize it may represent cells that undergo LPS-induced apoptosis [37–39].

Further analysis of dendritic cells using the Pareto archetype analysis approach may be promising due to the continuous nature of their gene expression, and their ability to perform multiple tasks. Future advances in single-cell RNA-Seq technology may allow better characterization of gene expression profiles of single cells from this fascinating population and achieve better understanding of their function. In addition, it would be highly interesting to

explore the spatial position of cells near the different archetypes in the spleen, which are known to contain DCs in different stages of their activation [24].

I. Uniformity of the distribution of cell states varies between tissues

Different tissues have different distributions of cell states, ranging from clumped to uniformly distributed geometries in gene expression space. Clumped geometry relates to the classic idea of separate cell types, where uniform filling challenges this view. Both geometries can be described by the Pareto perspective, with different distributions on the Pareto front – i.e. the polytope. Different factors that can influence this distribution are the shape of the fitness function, the shape of the performance functions where steep performance functions (as determined by a condition on their second derivative) lead to population of the front only near the vertices [5], and the flexibility that the system needs when facing a temporally fluctuating environment .

In this section, we quantify the deviation from a uniform distribution for each tissue. To do this, we compute the mean local density ρ_L in a ball of volume V around each data point, where V is equal to the volume of the convex hull of the data V_{CH} divided by the number of data points N. We then compute the same mean local density for a uniform distribution, ρ_U, by generating N uniformly distributed random points in the same convex hull. ρ_U is equal to the global density $\frac{N}{V_{CH}}$ with a correction for edge effects for points near the convex hull. The deviation from uniformity of the data is then given by:

$$\rho = \frac{\rho_L}{\rho_U}.$$

When the data is more clustered then uniform distribution, the average local density around data points is higher than for a uniform distribution, thus $\rho > 1$. The larger ρ is, the larger the deviation from uniform distribution. $\rho < 1$ reflects a deviation from a uniform distribution toward a more ordered geometry, e.g. if the points are arranged on a lattice. A value of $\rho = 1$ is consistent with a uniform distribution. This measure is inspired by Ripley's K function [40].

We projected the data points and the archetypes into the 3 first PCs space of the data, and the density analysis was carried in this space. Uniformly distributed random points were drawn 10 times for each datasets to estimate mean and error bars.

We found that bone marrow cells are the most clustered among the datasets we checked ($\rho = 4.67$) (S16 Figure), aligning with the classic view of the hierarchical hematopoietic lineage [41,42]. Intestinal cells are less clustered ($\rho = 2.93$), dendritic cells even more ($\rho = 2.42$), and intestinal progenitors are the most continuous ($\rho = 1.06$), in agreement with recent findings about their plasticity [43] and with studies on embryonic stem cells [44].

J. Comparison of archetypal analysis to Principal Component Analysis

In this section we compare archetypal analysis (AA) and Principal Component Analysis (PCA) as applied to the single cell intestinal dataset. A fair comparison is between 3PCs and a tetrahedron, because they both describe 3D subspaces of the 76D gene space. Another way to see this is that each point in the 3PC space is described by three coordinates, and each point in the tetrahedron is also described by three coordinates, due to the constraint $\sum_{i=1}^{N} \theta_i = 1$, where N is the number of archetypes, and θ_i are the weights of the archetypes $\vec{a}_i$ for each cell, $\vec{x}_{est} = \sum_{i=1}^{N} \theta_i \vec{a}_i$.

The AA result defines the data much more strictly than PCA: 3PCs allows any point in the 3D subspace spanned by the 3PCs, whereas AA restricts the data to be inside a tetrahedron, which is only a small part of the 3D space (S17 Figure).

Thus, the fact that a tetrahedron whose vertices are on the convex hull of the data explains almost all of the variance that is explained by the first 3 PCs (45%, 47%, respectively – i.e. 96% of the 3PC explained variance) is remarkable.

It is useful to compare the gene expression profiles that correspond to the first PCs to the gene expression profiles of the archetypes (S18 Figure). Figure S19 show the projections of the archetypes on the first 6 PCs to more clearly present these differences. It can be seen that the first PC is composed largely of archetype 3 (stem cells) minus archetype 1 (enterocytes); the second PC is composed of archetype 1 (enterocytes) minus archetype 2 (Nodal cells), while the third PC has the biggest contribution from the 4th archetype (goblet cells). PCs 4-6 do not correlate specifically with any of the archetypes.

In general, the PCs mix together different archetypes and thus it is harder to infer the extreme gene expressions of each cell type from them. This is because the tetrahedron is not aligned with the PC axes.

In addition to this analysis, we compared the sensitivity of the description by PCA to the sensitivity of the AA description, in a similar way to S1 Text Section C: "Comparison of archetypal analysis to clustering methods". We created 1,000 bootstrapped sets by sampling with replacement data points (the same bootstrapped sets that were used in S1 Text Section C). For each bootstrapped dataset we computed the positions of the 4 archetypes by PCHA algorithm and the coordinates of the 3 first PCs.

Each cell in each dataset was described by 76 coordinates. In archetypal analysis – the coordinates of the best description of the cell by linear combination of the archetypes:

$$\vec{x}_{est} = \sum_{i=1}^{N} \theta_i \vec{a}_i \ , \ \sum_{i=1}^{N} \theta_i = 1, \ \theta_i \in [0\ ,1],$$

Where $\vec{x}_{est}$ is the approximated description of data point (cell) $\vec{x}$, $\vec{a}_i$ are the archetypes vectors, and N=4 is the number of archetypes,

And in PCA – the projection of the cell on the first 3 PCs:

$\vec{x}_{est} = \sum_{i=1}^{N} \theta_i \overrightarrow{pc}_i, \quad \theta_i \in \mathbb{R}$,

Where N=3 is the number of archetypes, and $\overrightarrow{pc}_i$ are the PC vectors. Note that while the PCs have to be orthogonal, the archetype vectors do not obey this constraint.

We gathered these descriptions for each cell, for each of the methods. Then we computed the normalized standard deviation (SD) for each cell by dividing the standard deviation along the 1st principal component of variation in these descriptions by the standard deviation of the 1st principal component of the complete real data:

$$\sigma_{norm} = \frac{\sigma(1^{st}\ PC\ of\ \vec{x}_{est})}{\sigma(1^{st}\ PC\ of\ data)}$$

The results, presented in Figure S3, show that PCA is less sensitive to the sampling of the data than clustering techniques, but more sensitive than archetypal analysis. Most of the variation was due to lack of robustness of the third PC which separates the goblet cells. It appears that the relatively small number of goblet cells in the dataset caused large fluctuations in the third PC coordinates when bootstrapping, where archetypal analysis was able to sufficiently buffer these fluctuations presumably because it uses information distributed more uniformly across the entire dataset to infer archetype positions.

K. Increasing the number of archetypes may reveal subtle trends

In the manuscript, we chose to fit the intestinal cells data to a 3d-polytope, namely a tetrahedron. The reasons are described in *Methods: Determining the number of archetypes.*

However, further analysis using higher order polytopes may reveal more subtle trends. In Figure S20 we show a dendrogram-like tree of archetypes, in which we relate the archetypes found in different polytope fits. The tree was generated by fitting the data to k archetypes and computing their Euclidean distance, in the 76-dimensional gene expression space, from the k-1 archetypes whose positions were computed before. Characterization of these archetypes was then done by carrying a leave-1-out enrichment analysis (Methods: 1D Gene enrichment at archetypes), and inspecting the enriched genes. The enriched genes in each archetype for n = 2 to 6 is shown in Table S7.

It may be seen that with two archetypes (line), the separation between stem cells and enterocytes is captures. At three archetypes (triangle) the nodal class splits from the enterocytes. At four archetypes a goblet archetype appears, and remains distinct thereafter. At five archetypes, stem cells split into two archetypes. At six archetypes, nodal and enterocyte archetypes begin to mix.

References

1. Dalerba P, Kalisky T, Sahoo D, Rajendran PS, Rothenberg ME, Leyrat AA, et al. Single-cell dissection of transcriptional heterogeneity in human colon tumors. Nat Biotechnol. 2011;29: 1120–1127. doi:10.1038/nbt.2038

2. McDavid A, Finak G, Chattopadyay PK, Dominguez M, Lamoreaux L, Ma SS, et al. Data exploration, quality control and testing in single-cell qPCR-based gene expression experiments. Bioinformatics. 2013;29: 461–467. doi:10.1093/bioinformatics/bts714

3. McDavid A, Dennis L, Danaher P, Finak G, Krouse M, Wang A, et al. Modeling Bi-modality Improves Characterization of Cell Cycle on Gene Expression in Single Cells. PLoS Comput Biol. 2014;10: e1003696. doi:10.1371/journal.pcbi.1003696

4. Jain AK, Murty MN, Flynn PJ. Data Clustering: A Review. ACM Comput Surv. 1999;31: 264–323. doi:10.1145/331499.331504

5. Shoval O, Sheftel H, Shinar G, Hart Y, Ramote O, Mayo A, et al. Evolutionary Trade-Offs, Pareto Optimality, and the Geometry of Phenotype Space. Science. 2012;336: 1157–1160. doi:10.1126/science.1217405

6. MacQueen J. Some methods for classification and analysis of multivariate observations. The Regents of the University of California; 1967. Available: http://projecteuclid.org/euclid.bsmsp/1200512992

7. SOKAL R. A statistical method for evaluating systematic relationships. Univ Kans Sci Bull. 1958;38: 1409–1438.

8. Tamayo P, Slonim D, Mesirov J, Zhu Q, Kitareewan S, Dmitrovsky E, et al. Interpreting patterns of gene expression with self-organizing maps: Methods and application to hematopoietic differentiation. Proc Natl Acad Sci. 1999;96: 2907–2912. doi:10.1073/pnas.96.6.2907

9. Kohonen T. The self-organizing map. Proc IEEE. 1990;78: 1464–1480. doi:10.1109/5.58325

10. Morup M, Hansen LK. Archetypal analysis for machine learning. 2010 IEEE International Workshop on Machine Learning for Signal Processing (MLSP). 2010. pp. 172–177. doi:10.1109/MLSP.2010.5589222

11. Amir ED, Davis KL, Tadmor MD, Simonds EF, Levine JH, Bendall SC, et al. viSNE enables visualization of high dimensional single-cell data and reveals phenotypic heterogeneity of leukemia. Nat Biotechnol. 2013;31: 545–552. doi:10.1038/nbt.2594

12. Lee M-CW, Lopez-Diaz FJ, Khan SY, Tariq MA, Dayn Y, Vaske CJ, et al. Single-cell analyses of transcriptional heterogeneity during drug tolerance transition in cancer cells by RNA sequencing. Proc Natl Acad Sci. 2014;111: E4726–E4735. doi:10.1073/pnas.1404656111

13. Blainey PC, Quake SR. Dissecting genomic diversity, one cell at a time. Nat Methods. 2014;11: 19–21. doi:10.1038/nmeth.2783

14. Patel AP, Tirosh I, Trombetta JJ, Shalek AK, Gillespie SM, Wakimoto H, et al. Single-cell RNA-seq highlights intratumoral heterogeneity in primary glioblastoma. Science. 2014;344: 1396–1401. doi:10.1126/science.1254257

15. Burrell RA, McGranahan N, Bartek J, Swanton C. The causes and consequences of genetic heterogeneity in cancer evolution. Nature. 2013;501: 338–345. doi:10.1038/nature12625

16. Bendall SC, Simonds EF, Qiu P, Amir ED, Krutzik PO, Finck R, et al. Single-Cell Mass Cytometry of Differential Immune and Drug Responses Across a Human Hematopoietic Continuum. Science. 2011;332: 687–696. doi:10.1126/science.1198704

17. Banchereau J, Briere F, Caux C, Davoust J, Lebecque S, Liu YJ, et al. Immunobiology of dendritic cells. Annu Rev Immunol. 2000;18: 767–811. doi:10.1146/annurev.immunol.18.1.767

18. Amit I, Garber M, Chevrier N, Leite AP, Donner Y, Eisenhaure T, et al. Unbiased reconstruction of a mammalian transcriptional network mediating the differential response to pathogens. Science. 2009;326: 257–263. doi:10.1126/science.1179050

19. Merad M, Sathe P, Helft J, Miller J, Mortha A. The dendritic cell lineage: ontogeny and function of dendritic cells and their subsets in the steady state and the inflamed setting. Annu Rev Immunol. 2013;31: 563–604. doi:10.1146/annurev-immunol-020711-074950

20. Shortman K, Liu Y-J. Mouse and human dendritic cell subtypes. Nat Rev Immunol. 2002;2: 151–161. doi:10.1038/nri746

21. Collin M, McGovern N, Haniffa M. Human dendritic cell subsets. Immunology. 2013;140: 22–30. doi:10.1111/imm.12117

22. Shalek AK, Satija R, Adiconis X, Gertner RS, Gaublomme JT, Raychowdhury R, et al. Single-cell transcriptomics reveals bimodality in expression and splicing in immune cells. Nature. 2013;498: 236–240. doi:10.1038/nature12172

23. Hey YY, O'Neill HC. Murine spleen contains a diversity of myeloid and dendritic cells distinct in antigen presenting function. J Cell Mol Med. 2012;16: 2611–2619. doi:10.1111/j.1582-4934.2012.01608.x

24. Mildner A, Jung S. Development and Function of Dendritic Cell Subsets. Immunity. 2014;40: 642–656. doi:10.1016/j.immuni.2014.04.016

25. Jaitin DA, Kenigsberg E, Keren-Shaul H, Elefant N, Paul F, Zaretsky I, et al. Massively Parallel Single-Cell RNA-Seq for Marker-Free Decomposition of Tissues into Cell Types. Science. 2014;343: 776–779. doi:10.1126/science.1247651

26. Subramanian A, Tamayo P, Mootha VK, Mukherjee S, Ebert BL, Gillette MA, et al. Gene set enrichment analysis: A knowledge-based approach for interpreting genome-wide

expression profiles. Proc Natl Acad Sci. 2005;102: 15545–15550. doi:10.1073/pnas.0506580102

27. Hart Y, Sheftel H, Hausser J, Szekely P, Ben-Moshe NB, Korem Y, et al. Inferring biological tasks using Pareto analysis of high-dimensional data. Nat Methods. 2015;advance online publication. doi:10.1038/nmeth.3254

28. Edelaar P. Comment on “Evolutionary Trade-Offs, Pareto Optimality, and the Geometry of Phenotype Space.” Science. 2013;339: 757–757. doi:10.1126/science.1228281

29. Shoval O, Sheftel H, Shinar G, Hart Y, Ramote O, Mayo A, et al. Response to Comment on “Evolutionary Trade-Offs, Pareto Optimality, and the Geometry of Phenotype Space.” Science. 2013;339: 757–757. doi:10.1126/science.1228921

30. Condamine T, Le Texier L, Howie D, Lavault A, Hill M, Halary F, et al. Tmem176B and Tmem176A are associated with the immature state of dendritic cells. J Leukoc Biol. 2010;88: 507–515. doi:10.1189/jlb.1109738

31. Jang MH, Sougawa N, Tanaka T, Hirata T, Hiroi T, Tohya K, et al. CCR7 Is Critically Important for Migration of Dendritic Cells in Intestinal Lamina Propria to Mesenteric Lymph Nodes. J Immunol. 2006;176: 803–810. doi:10.4049/jimmunol.176.2.803

32. Kiama SG, Cochand L, Karlsson L, Nicod LP, Gehr P. Evaluation of phagocytic activity in human monocyte-derived dendritic cells. J Aerosol Med Off J Int Soc Aerosols Med. 2001;14: 289–299. doi:10.1089/089426801316970240

33. Nagl M, Kacani L, Müllauer B, Lemberger E-M, Stoiber H, Sprinzl GM, et al. Phagocytosis and Killing of Bacteria by Professional Phagocytes and Dendritic Cells. Clin Diagn Lab Immunol. 2002;9: 1165–1168. doi:10.1128/CDLI.9.6.1165-1168.2002

34. Steinman RM, Nussenzweig MC. Avoiding horror autotoxicus: The importance of dendritic cells in peripheral T cell tolerance. Proc Natl Acad Sci U S A. 2002;99: 351–358. doi:10.1073/pnas.231606698

35. Banchereau J, Steinman RM. Dendritic cells and the control of immunity. Nature. 1998;392: 245–252. doi:10.1038/32588

36. Foti M, Granucci F, Aggujaro D, Liboi E, Luini W, Minardi S, et al. Upon dendritic cell (DC) activation chemokines and chemokine receptor expression are rapidly regulated for recruitment and maintenance of DC at the inflammatory site. Int Immunol. 1999;11: 979–986. doi:10.1093/intimm/11.6.979

37. Kushwah R, Hu J. Dendritic Cell Apoptosis: Regulation of Tolerance versus Immunity. J Immunol. 2010;185: 795–802. doi:10.4049/jimmunol.1000325

38. Zanoni I, Ostuni R, Capuano G, Collini M, Caccia M, Ronchi AE, et al. CD14 regulates the dendritic cell life cycle after LPS exposure through NFAT activation. Nature. 2009;460: 264–268. doi:10.1038/nature08118

39. Leverkus M, McLellan AD, Heldmann M, Eggert AO, Bröcker E-B, Koch N, et al. MHC class II-mediated apoptosis in dendritic cells: a role for membrane-associated and

mitochondrial signaling pathways. Int Immunol. 2003;15: 993–1006. doi:10.1093/intimm/dxg099

40. Ripley BD. Modelling spatial patterns. J R Stat Soc. 1977;B39: 172–212.

41. Chao MP, Seita J, Weissman IL. Establishment of a Normal Hematopoietic and Leukemia Stem Cell Hierarchy. Cold Spring Harb Symp Quant Biol. 2008; sqb.2008.73.031. doi:10.1101/sqb.2008.73.031

42. Phillips RL, Ernst RE, Brunk B, Ivanova N, Mahan MA, Deanehan JK, et al. The Genetic Program of Hematopoietic Stem Cells. Science. 2000;288: 1635–1640. doi:10.1126/science.288.5471.1635

43. Blanpain C, Fuchs E. Plasticity of epithelial stem cells in tissue regeneration. Science. 2014;344: 1242281. doi:10.1126/science.1242281

44. Hough SR, Laslett AL, Grimmond SB, Kolle G, Pera MF. A continuum of cell states spans pluripotency and lineage commitment in human embryonic stem cells. PloS One. 2009;4: e7708. doi:10.1371/journal.pone.0007708
